# Supplementary material for: HiC-DC+ enables systematic 3D interaction calls and differential analysis for Hi-C and HiChIP
Source: Nat Commun. 2021 Jun 7;12:3366. doi: 10.1038/s41467-021-23749-x (PMC8184932; doi:10.1038/s41467-021-23749-x)
Supplement: Supplementary file 1 — Supplementary Information [file 41467_2021_23749_MOESM1_ESM.pdf]

Supplementary Note 1. We do not use ChIP signal (or HiChIP self-ligation signal as a proxy for ChIP) in order to normalize HiChIP interaction calls. We hypothesized that explicitly modeling ChIP enrichment may *decrease* the power to detect interactions or differential interactions by normalizing away the true signal. To test our claim, we modeled ChIP enrichment explicitly at 5kb by adding any of the following covariates to the model for K562 H3K27ac HiChIP data:

- chip: standardized log transformed H3K27ac ChIP intensity
- cov_counts: interaction bin counts divided by the average count over bins that share the same peak status (i.e., both anchors have a peak (peak-to-peak), one has a peak (peak-to-all), neither anchor has peak (all-to-all)), similar to FitHiChIP
- peak: peak status as described above as a categorical covariate with three levels
- cov: standardized log transformed ChIP enrichment level measured by the number of short-range reads (intra-chromosomal reads ≤ 1kb), similar to MAPS

Then, we tested the performance of these two models on K562 CRISPRi-FlowFISH data by per gene auPR values (Supplementary Fig. 8; overall auPR of 0.205 for HiC-DC+ vs. 0.054 for HiC-DC+ (chip), 0.124 for HiC-DC+ (cov_counts), 0.128 for HiC-DC+ (peak), and 0.051 for HiC-DC+ (cov)). Including ChIP enrichment as a covariate worsens the performance for detection of enhancer-promoter interactions from CRISPRi-FlowFISH data (Supplementary Fig. 8, $P < 0.05$, Wilcoxon signed-rank test).

Supplementary Note 2. We were curious to see how H3K27ac HiChIP interaction calls performed relative to the "Activity-by-Contact" (ABC) model for predicting promoter-enhancer loops. The ABC model scores the effect of a putative regulatory element on a gene promoter by taking the product of the enhancer's activity (A), defined as the geometric mean of DNase-seq and H3K27ac ChIP-seq read counts, and the KR normalized Hi-C contact frequency (C) between the element and promoter; the raw score is normalized by the sum of A times C for all accessible elements within 5 Mb of the promoter. We found that the ABC score outperformed HiC-DC+ HiChIP calls when evaluated by auPR per gene (Supplementary Fig. 11). Interestingly, an "Activity-by-Distance" (ABD) score, simply defined by product of the inverse genomic distance with normalized activity, significantly outperforms the ABC score by this measure. Since ABD uses neither Hi-C nor HiChIP, these results suggest that the promoter-enhancer interactions identified by CRISPRi-FlowFISH are enriched for shorter-range interactions that are detectable by H3K27ac signal and accessibility alone. Despite this caveat, enhancer screening data provides a useful benchmark data set for comparing HiChIP interaction callers.

Supplementary Note 3. To see whether our differential HiChIP interactions are mostly driven by ChIP enrichment bias or underlying chromatin architecture changes, we found differential H3K27ac HiChIP interactions (FDR < 0.05 and |logFC| >1) at 5kb between K562 and GM12878 and compared to Hi-C data in the same cell lines[1, 2] as well as differential H3K27ac ChIP-seq peaks (FDR < 0.05). As we do not have Hi-C replicates of similar coverage for GM12878 and K562, we used the change in normalized Hi-C signal (O/E) from HiC-DC+ as a proxy for differential signal. We found that gained/lost HiChIP interactions are closely associated with increased/decreased Hi-C signal as well as increased/decreased ChIP signal in at least one of the anchors (Supplementary Fig. 24). This suggests that differential HiChIP interactions are driven not only by ChIP but also by Hi-C signal. To further support our claim, we grouped differential HiChIP interactions based on whether none, one, or both anchors overlap with differential ChIP-seq peaks:

- chip_sig_sig: both anchors have an overlapping differential ChIP-seq peak
- chip_sig_stable: one of the anchors has an overlapping differential ChIP-seq peak, the other anchor does not
- chip_stable: neither of the anchors has such an overlapping differential ChIP-seq peak

We further separated each of these three sets of interactions into whether the ChIP-seq peaks overlapping with the associated anchors had exhibited a positive log-fold change (“up”) or a negative log-fold change (“down”) on average.

Supplementary Fig. 30a shows the change in normalized Hi-C (O/E) counts for each of these 6 categories in lost H3K27ac HiChIP interactions in K562 over GM12878, whereas Supplementary Fig. 30b shows the respective change in gained H3K27ac HiChIP interactions in K562 over GM12878. Note, as expected, that the sign of the average ChIP-seq signal change in the anchors (blue vs. red bars in Supplementary Fig. 30a,b) largely coincided with the sign of the HiChIP interaction (e.g. almost all chip_sig_sig interactions that are lost in K562 vs. GM12878 also lose ChIP-seq signal). Importantly, most of our differential HiChIP interactions exhibit change in Hi-C interactions in the same direction independent of the ChIP status. Indeed, the differential HiChIP interactions with the strongest concordant ChIP signal changes (chip_sig_sig) had the largest differential Hi-C signal as well. Rather than identifying differential HiChIP interactions solely due to ChIP signal, we found that the majority (61%) of HiC-DC+ differential HiChIP interactions with differential ChIP-seq signal at one or more anchors were supported by a concordant differential Hi-C signal ($|\log2FC$ of normalized counts$| \geq 0.58$; i.e., $FC \geq 1.5$ or $FC \leq 0.67$).

Conversely, we also found some (27%) differential HiChIP interactions with small changes in Hi-C ($|\log2FC$ of normalized counts$| < 0.58$; i.e., $0.67 < FC < 1.5$) but substantial changes in ChIP intensity. We believe that these differential interactions could include real enhancer-promoter interactions that are not captured with high sensitivity via Hi-C. Indeed, we found such differential HiChIP interactions linked to a promoter in one anchor with small changes in Hi-C but substantial changes in ChIP intensity in the other anchor are depleted in significant Hi-C interactions (odds ratio=0.71, $P < 1.0 \times 10^{-16}$, Fisher’s exact test) and enriched in differentially expressed genes (odds ratio=1.11, $P < 7 \times 10^{-3}$,Fisher’s exact test) compared to other HiChIP interactions linked to a promoter. IGV tracks in Supplementary Fig. 25 show an example locus where Hi-C interactions do not change between cell lines while there are substantial changes in ChIP-seq and gene expression along with HiChIP interactions. It seems that Hi-C interactions in both cell lines are enriched around subTAD boundaries, whereas HiChIP interactions can efficiently reveal cell line specific promoter-anchored interactions for genes that are also exclusively expressed in that cell line.

Function	Description	Time (min)	Memory (GB)	Number of processors
construct_features	Generates features for each bin	2.96	0.53	1
construct_features_parallel	Generates features for each bin	0.90	0.52	8
generate_bintolen_gi_list	Generates data storage matrix with features	0.92	3.13	1
add_hicpro_allvalidpairs_counts	Adds counts to the data storage matrix	7.63	5.86	1
expand_1D_features	Expands features to each 2D bin	0.82	10.9	1
HiCDCPlus	Runs the statistical model to find significant interactions	5.49	17.1	1
HiCDCPlus_parallel	Runs the statistical model to find significant interactions	2.84	17.1	8
gi_list_write	Outputs the results to a txt file	0.69	17.1	1
Total		18.51	17.1	1
Total parallel (8 cores)		13.34	17.1	8

Supplementary Table 1. Time and memory usage of various HiC-DC+ functions utilizing mESC HiChIP allvalidPairs (367.9 million all valid pairs) to find significant interactions at 5kb resolution on Mac (2.4 GHz, 8 core i9, 64 GB memory) R version (4.0.3), OS version 11.1.

Function	Description	Time (min)	Memory (GB)	Number of processors
hicdcdiff, diagnostics=F	Finds differential interactions without diagnostic plots	1.72	0.66	1
hicdcdiff, diagnostics=T	Finds differential interactions with diagnostic plots	2.55	0.67	1

Supplementary Table 2. Time and memory usage of various HiC-DC+ functions utilizing mESC HiChIP .hic files (2 files representing 2 replicates) and MEF HiChIP .hic files (2 files representing 2 replicates) to find differential HiChIP interactions at 5kb resolution on Mac (2.4 GHz, 8 core i9, 64 GB memory) R version (4.0.3), OS version 11.1.

Function	Description	Time (min)	Memory (GB)	Number of processors
construct_features	Generates features for each bin	3.49	0.53	1
construct_features_parallel	Generates features for each bin	1.15	0.52	8
generate_bintolen_gi_list	Generates data storage matrix with features	1.08	3.52	1
add_hic_counts	Adds counts to the data storage matrix	14.91	5.64	1
expand_1D_features	Expands features to each 2D bin	1.78	11.4	1
HiCDCPlus	Runs the statistical model to find significant interactions	7.49	17.8	1
HiCDCPlus_parallel	Runs the statistical model to find significant interactions	4.19	17.8	8
gi_list_write	Outputs the results to a txt file	1.32	17.8	1
Total		30.08	17.8	1
Total parallel (8 cores)		23.72	17.8	8

Supplementary Table 3. Time and memory usage of various HiC-DC+ functions utilizing GM12878 Hi-C .hic file (Rao et al., 2014, 3.68 billion reads) to find significant interactions at 5kb resolution on Mac (2.4 GHz, 8 core i9, 64 GB memory) R version (4.0.3), OS version 11.1.

Function	Description	Time (min)	Memory (GB)	Number of processors
construct_features	Generates features for each bin	2.87	0.48	1
construct_features_parallel	Generates features for each bin	0.84	0.48	8
generate_bintolen_gi_list	Generates data storage matrix with features	0.90	3.06	1
add_hicpro_allvalidpairs_counts	Adds counts to the data storage matrix	7.67	5.75	1
expand_1D_features	Expands features to each 2D bin	0.82	10.8	1
HiCDCPlus	Runs the statistical model to find significant interactions	10.53	17.0	1
HiCDCPlus_parallel	Runs the statistical model to find significant interactions	3.19	17.0	8
gi_list_write	Outputs the results to a txt file	0.39	17.0	1
Total		23.20	17.0	1
Total parallel (8 cores)		13.36	17.0	8

Supplementary Table 4. Time and memory usage of various HiC-DC+ functions utilizing mESC HiChIP allvalidPairs (367.9 million all valid pairs) to find significant interactions at 5kb resolution on Linux (CentOS 7 HPC cluster with LSF queuing system, 2.3 GHz) R version 3.6.1.

Function	Description	Time (min)	Memory (GB)	Number of processors
construct_features	Generates features for each bin	3.03	0.48	1
construct_features_parallel	Generates features for each bin	0.86	0.48	8
generate_bintolen_gi_list	Generates data storage matrix with features	0.96	3.44	1
add_hic_counts	Adds counts to the data storage matrix	13.67	5.53	1
expand_1D_features	Expands features to each 2D bin	1.15	11.3	1
HiCDCPlus	Runs the statistical model to find significant interactions	9.70	17.7	1
HiCDCPlus_parallel	Runs the statistical model to find significant interactions	4.78	17.7	8
gi_list_write	Outputs the results to a txt file	0.55	17.7	1
Total		29.05	17.7	1
Total parallel (8 cores)		22.29	17.7	8

Supplementary Table 5. Time and memory usage of various HiC-DC+ functions utilizing GM12878 Hi-C .hic file (Rao et al., 2014, 3.68 billion reads) to find significant interactions at 5kb resolution on Linux (CentOS 7 HPC cluster with LSF queuing system, 2.3 GHz) R version 3.6.1.

Datasets	Resolution	Number of significant interactions (FDR < 0.05)	Method
K562 H3K27ac HiChIP	5kb	768,629	HiC-DC+
K562 H3K27ac HiChIP	5kb	1,226,277	FitHiChIP (L)
K562 H3K27ac HiChIP	5kb	313,007	MAPS
K562 H3K27ac HiChIP	5kb	484804 (PET ≥ 2)	hichipper
GM12878 SMC HiChIP	5kb	81,861 (FDR < 0.01)	HiC-DC+
GM12878 SMC HiChIP	5kb	95,196 (FDR < 0.01)	FitHiChIP (L)
GM12878 SMC HiChIP	5kb	38,894 (FDR < 0.01)	MAPS
GM12878 SMC HiChIP	5kb	52,590 (PET ≥ 2)	hichipper
mESC H3K27ac HiChIP	5kb	173,618	HiC-DC+
mESC H3K27ac HiChIP	5kb	74,813	FitHiChIP (L)
THP-1 monocyte Hi-C	5kb	748,540	HiC-DC+
THP-1 macrophage Hi-C	5kb	832,902	HiC-DC+

Supplementary Table 6. Number of significant interactions detected by each method for various datasets.

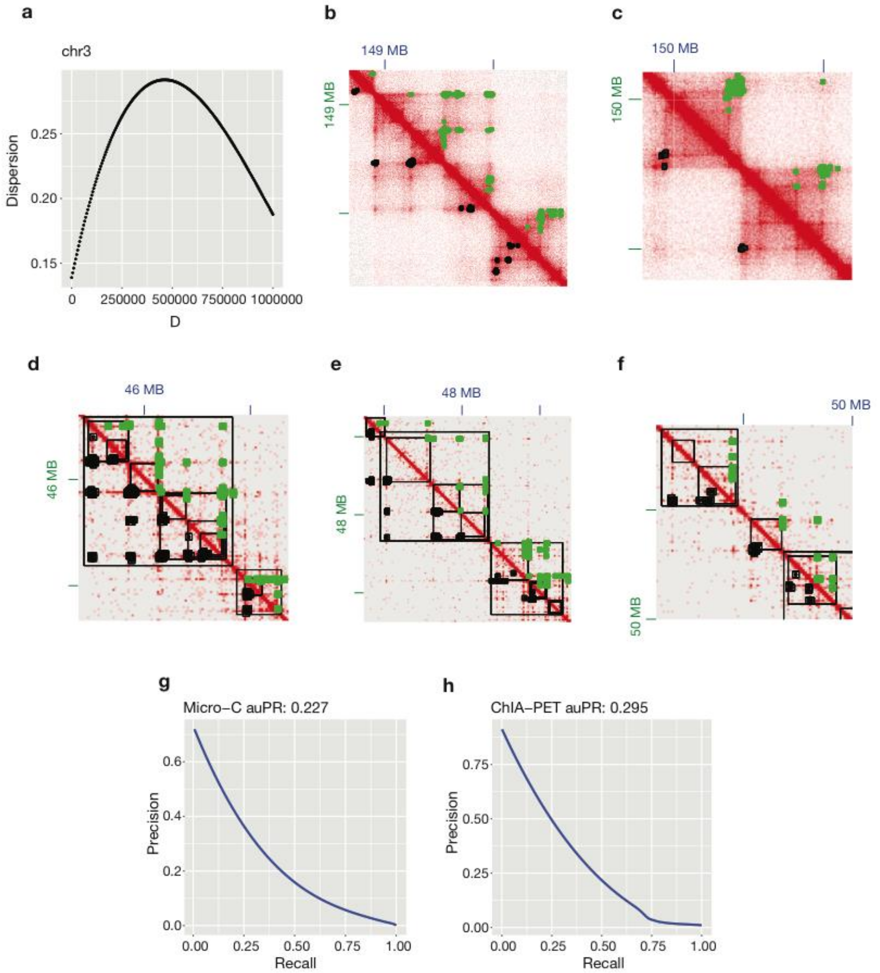

Supplementary Figure 1. HiC-DC+ interactions called on Micro-C data in H1-hESC from Krietenstein et al.[3] (4DNFI2TK7L2F) and CTCF ChIA-PET from 4DN (4DNFIDCEC4PQ). **a.** Dispersion parameter as a function of genomic distance found using Micro-C data for chromosome 3. We partitioned genomic distance into 50kb intervals and estimated a separate dispersion parameter for each these intervals by training HiC-DC+. We applied a third degree polynomial fit to the dispersion parameters and obtained the dispersion estimates for each distance band in the data using this fitted polynomial. **b,c.** As dispersion estimates varies greatly for Micro-C, we trained a negative binomial GLM with variable dispersion instead of the default fixed dispersion using GC content, mappability and distance dependence as covariates at 5kb. We overlaid HiCCUPS loops (black, below the diagonal) that we call using Hi-C data in H1-hESC (merged replicates of 4DNESRJ8KV4Q) with our HiC-DC+ interactions (green, above the diagonal) for chromosome 3. **d,e,f.** ChIA-PET maps have an abundance of zero count bins, thus we trained hurdle regression with distance dependence as power decay, GC content and mappability at 10kb as covariates for ChIA-PET. We overlaid HiCCUPS loops (black, below the diagonal) that we call using Hi-C data in GM12878 (merged replicates of 4DNFI1UEG1HD) with our HiC-DC+ interactions (green, above the diagonal) for chromosome 3. Most of the HiCCUPS interactions (~80%) overlap with HiC-DC+ interactions. Black squares represent Arrowhead subTAD annotations (GSE63525). 44% of HiC-DC+ and 45% of HiCCUPS loops were found at these subTAD corners. **g,h.** Precision-recall curves using HiCCUPS loops as labels for Micro-C and ChIA-PET, respectively. Respective auPR values are reported at the top.

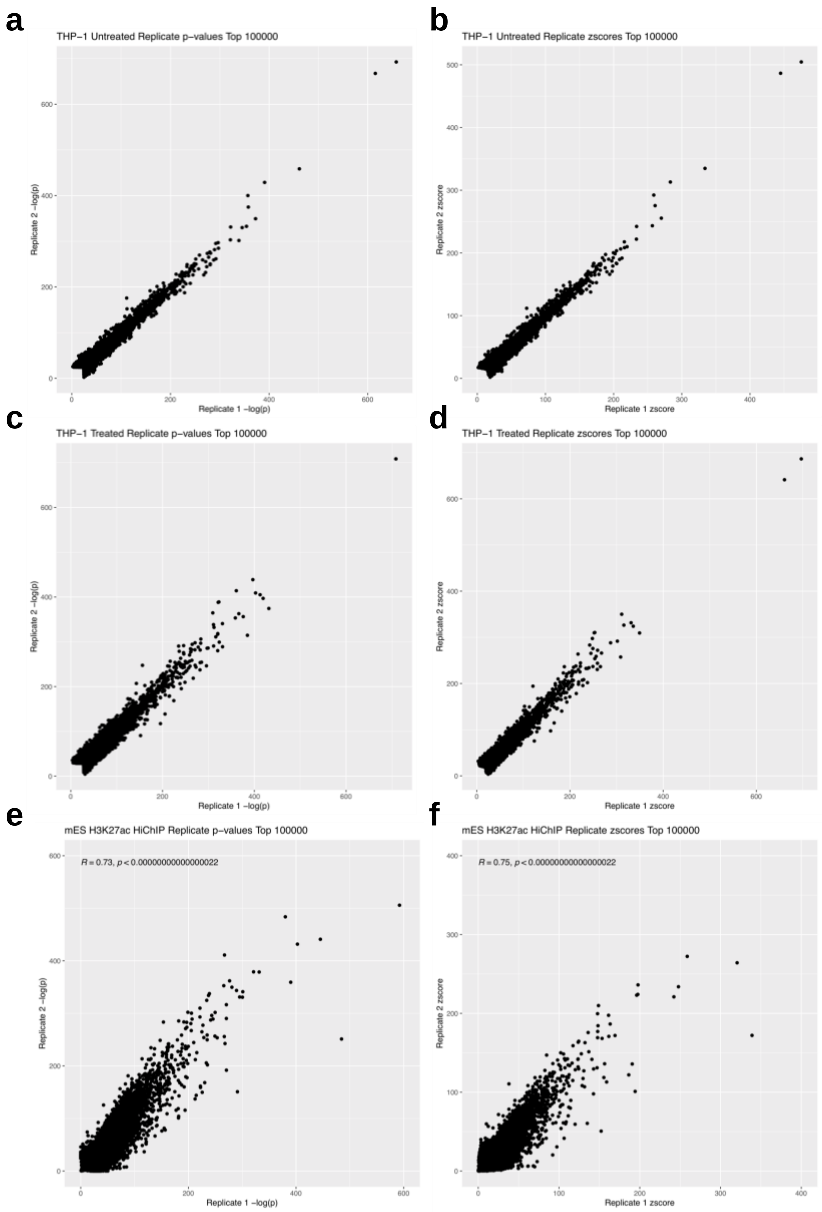

Supplementary Figure 2. Concordance of replicate -log10(*P*) and Z-scores estimated by HiC-DC+ in Hi-C and HiChIP. Scatter plots of replicate **a.** -log10(*P*) and **b.** Z-scores for top 100,000 interactions in untreated THP-1 (PRJNA385337), **c.** -log10(*P*) and **d.** Z-scores for top 100,000 interactions in treated THP-1 (PRJNA385337), **e.** -log10(*P*) and **f.** Z-scores for top 100,000 interactions in mESC H3K27ac HiChIP (GSM2705031, GSM2705032, GSM2705033, GSM2705034) denoted with the Spearman correlation coefficient. We also performed overlap analysis at 5kb resolution and found that 80%, 81%, and 63% of interactions identified in the respective primary replicates of untreated THP-1, treated THP-1 Hi-C and mES H3K37ac HiChIP were also called in the secondary replicates, reflecting high reproducibility.

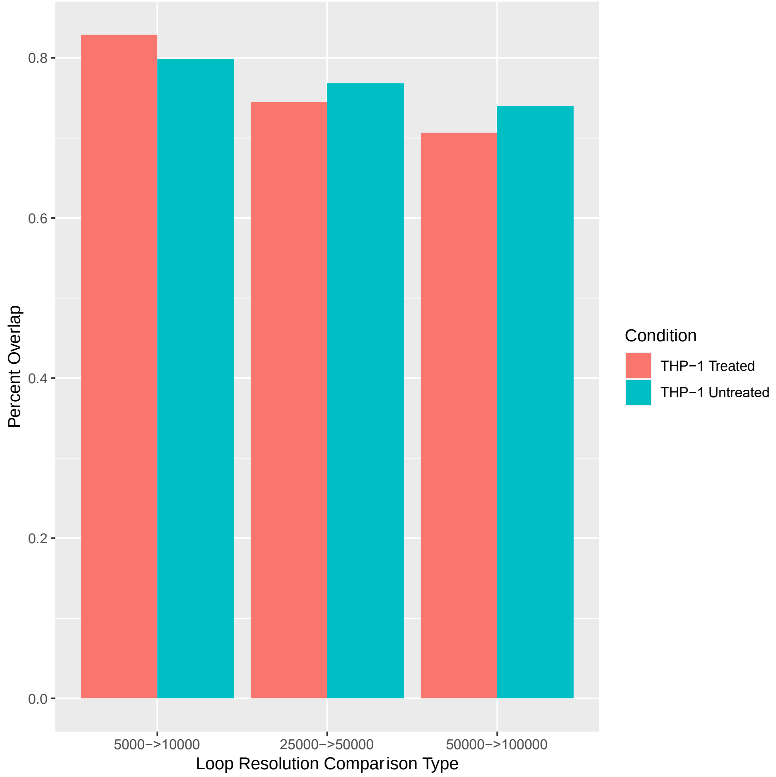

Supplementary Figure 3. Percent overlap of significant HiC-DC+ interactions in untreated and treated THP-1 Hi-C (PRJNA385337) in two consecutive resolutions.

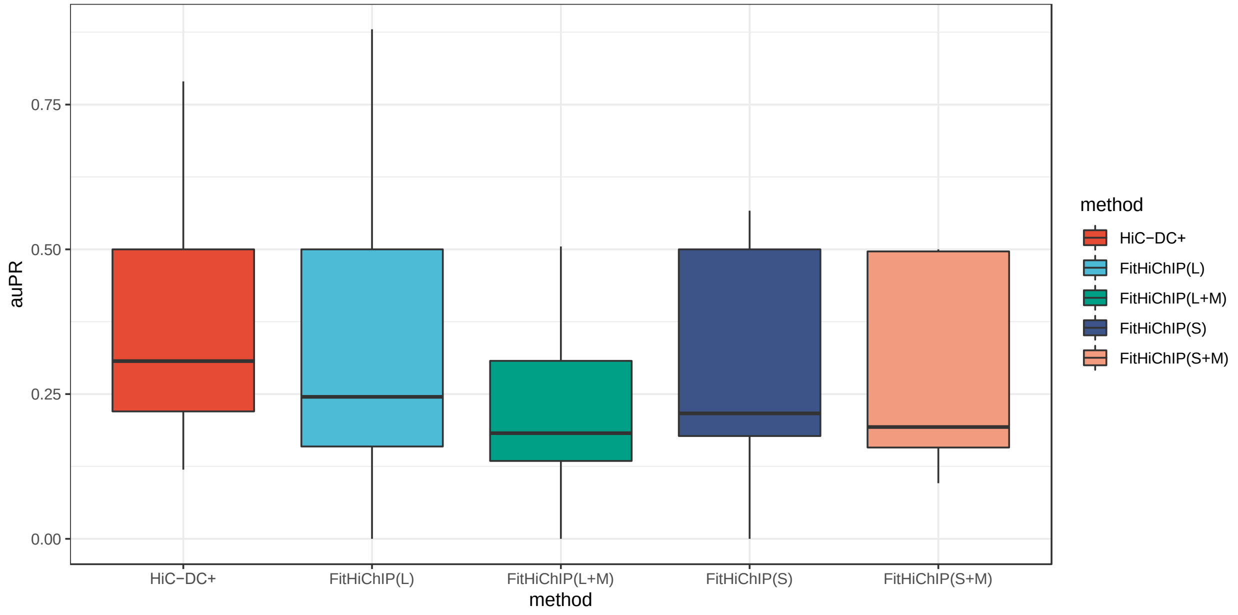

Supplementary Figure 4. Performance evaluation of HiC-DC+ and FitHiChIP variants by per gene auPR values. For each target gene, we ranked the candidate regulatory elements tested by CRISPRi-FlowFISH in K562 cells based on the significance (*P* value) of the HiChIP (merged replicates of GSM2705043, GSM2705044, GSM2705045) interactions with the promoter as estimated by each method (n=22 genes; Methods). Centers of the boxes indicate median values, the lower and upper hinges correspond to the first and third quartiles, and the upper (lower) whiskers extend from the hinge to the largest (smallest) value no further than 1.5 times the distance between the first and third quartiles.

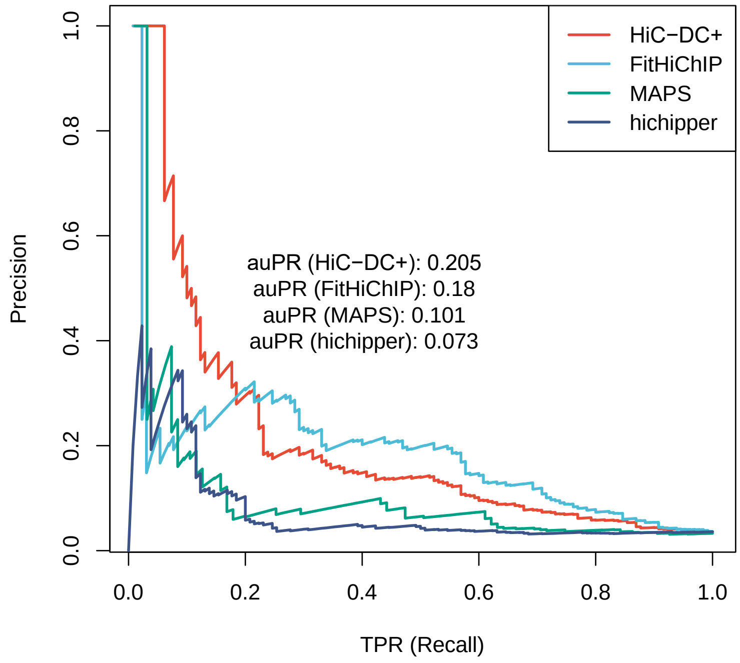

Supplementary Figure 5. Precision-recall curves for different methods on the data used to generate Fig. 2a.

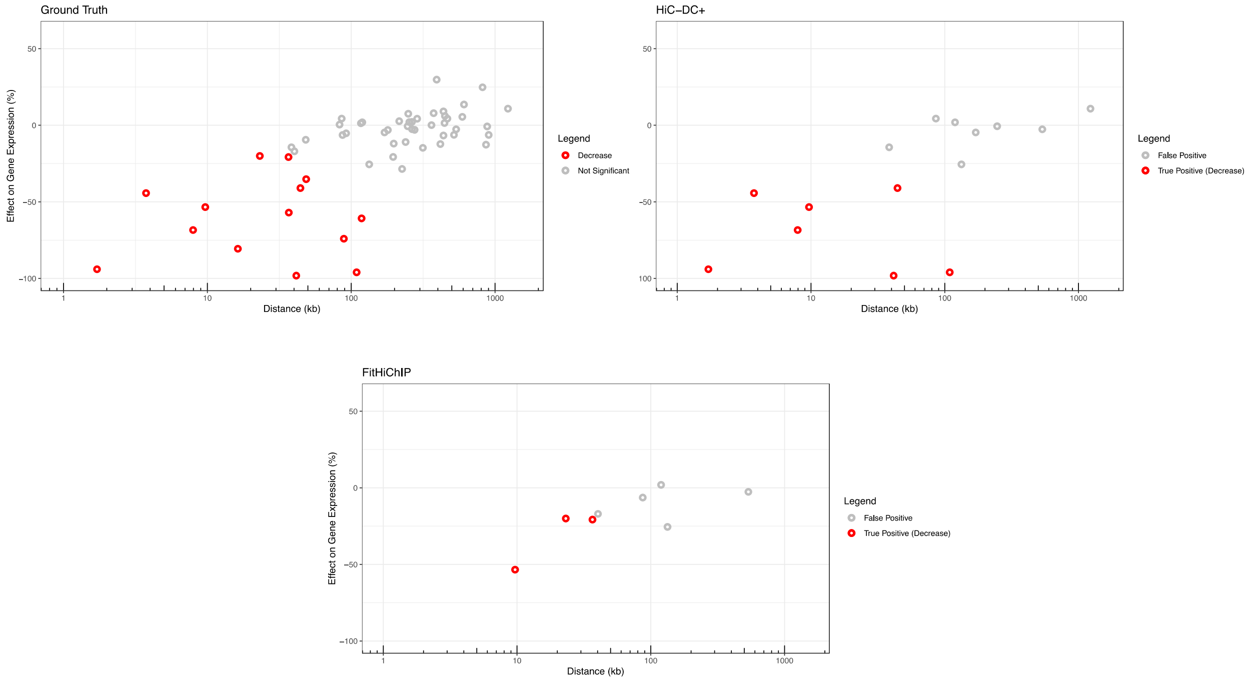

Supplementary Figure 6. Comparison of performance for identifying promoter-enhancer interactions in mES H3K27ac HiChIP (merged replicates of GSM2705031, GSM2705032, GSM2705033, GSM2705034) with large effect sizes on target gene expression as assessed by CRISPRi data in mESC data from Fulco et al.[4] (n=57 candidate pairs). Each dot in the scatterplots represents one tested promoter-enhancer pair and found to be significant at 1% FDR by the respective method. For FitHiChIP, we used mESC H3K27ac ChIP-seq peaks from ENCODE (ENCFF001XZL, n=12,682).

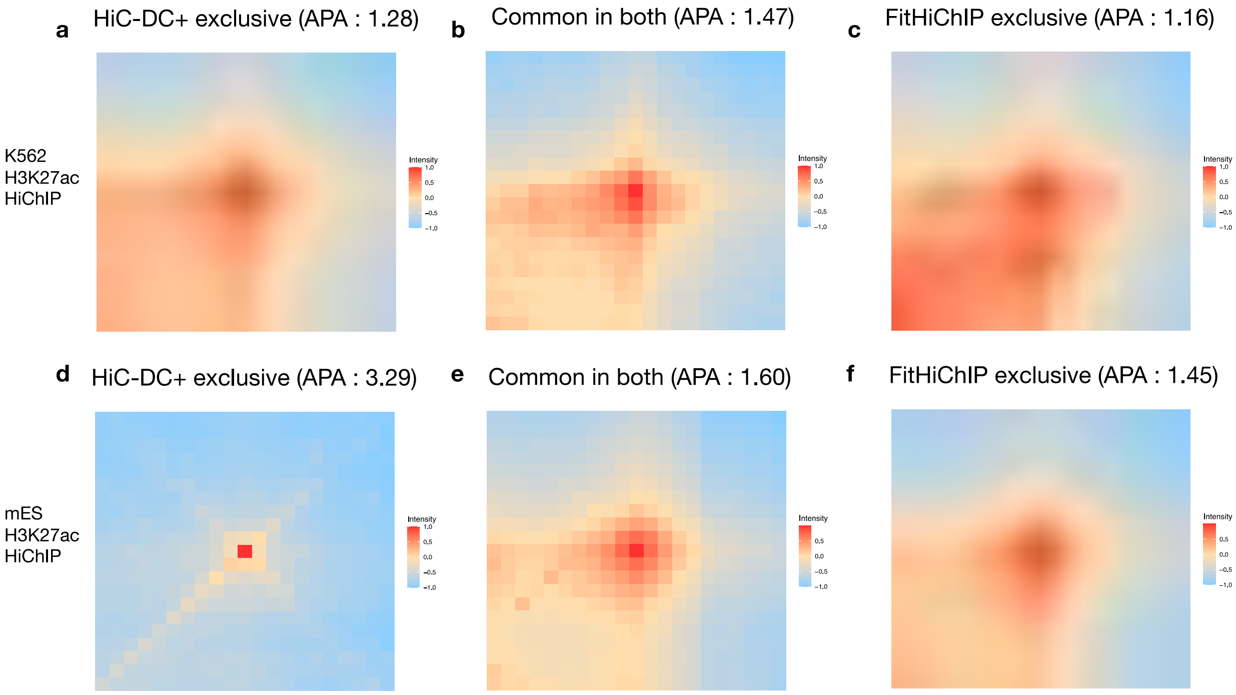

Supplementary Figure 7. APA plots for HiChIP interactions that are unique to HiC-DC+ (**a**,**d**) and FitHiChIP (**c**, **f**) and shared between HiC-DC+ and FitHiChIP (**b**,**e**). We generated APA plots of top 5000 significant interactions called by each method in mES H3K27ac (merged replicates of GSM2705031, GSM2705032, GSM2705033, GSM2705034) and K562 H3K27ac HiChIP (merged replicates of GSM2705043, GSM2705044, GSM2705045) data at 5kb resolution, using corresponding Hi-C data in mES (Bonev et al., 2017) (merged replicates of GSM2533818, GSM2533819, GSM2533820, GSM2533821) and K562 (Rao et al., 2014) (merged replicates of GSM1551618, GSM1551619, GSM1551620, GSM1551621, GSM1551622, GSM1551623) (Methods). Number of loops that passed APA filtering is: 2,311 for **(a)**; 211 for **(b)**; 52 for **(c)**; 1,111 for **(d)**; 457 for **(e)**; 900 for **(f).**

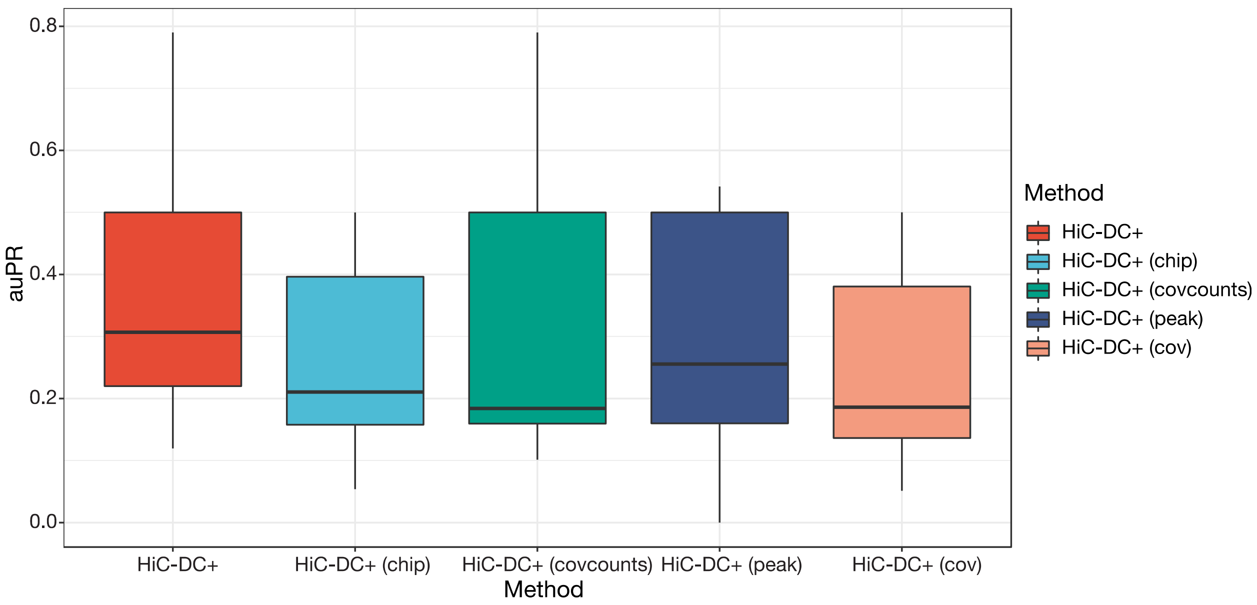

Supplementary Figure 8. Evaluation of HiC-DC+ and its variants incorporating ChIP intensity as a covariate (Supplementary Note 1) by per gene auPR values. For each target gene, we ranked the candidate regulatory elements tested by CRISPRi-FlowFISH in K562 cells based on the significance (*P* value) of the HiChIP (merged replicates of GSM2705043, GSM2705044, GSM2705045) interactions with the promoter as estimated by each method (n=22 genes; Methods). Centers of the boxes indicate median values, the lower and upper hinges correspond to the first and third quartiles, and the upper (lower) whiskers extend from the hinge to the largest (smallest) value no further than 1.5 times the distance between the first and third quartiles.

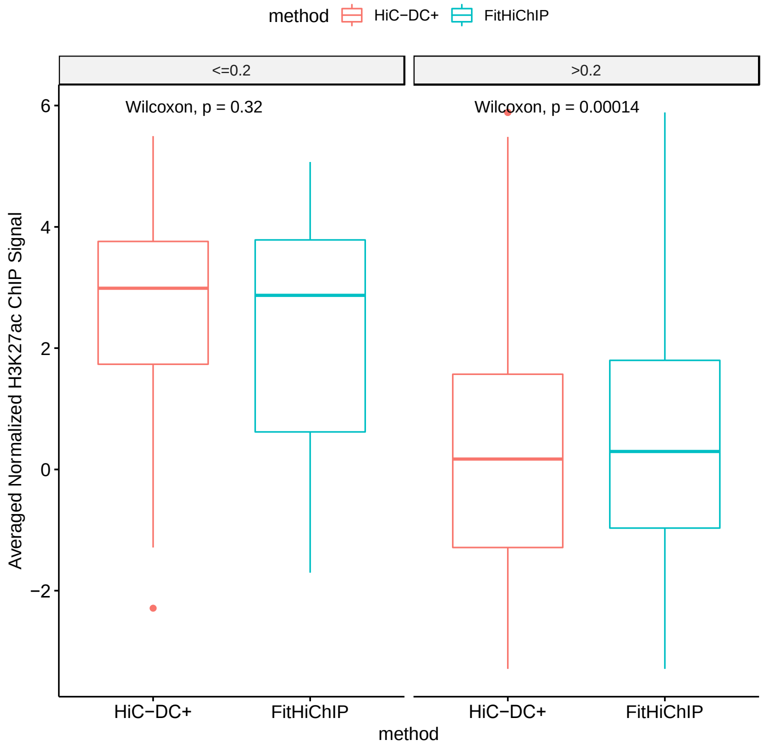

Supplementary Figure 9. Distribution of H3K27ac ChIP signal in candidate enhancer anchors of pairs found significant by either HiC-DC+ or FitHiChIP using K562 H3K27ac HiChIP (merged replicates of GSM2705043, GSM2705044, GSM2705045) at recall values lower or higher than 0.2 (*P* values reported based on two-sided Wilcoxon tests). Number of interactions at recall values less than or equal to 0.2 are 88 and 84 for HiC-DC+ and FitHiChIP, respectively. Number of interactions at recall values higher than 0.2 are 3530 and 3534 for HiC-DC+ and FitHiChIP. Centers of the boxes indicate median values, the lower and upper hinges correspond to the first and third quartiles, and the upper (lower) whiskers extend from the hinge to the largest (smallest) value no further than 1.5 times the distance between the first and third quartiles.

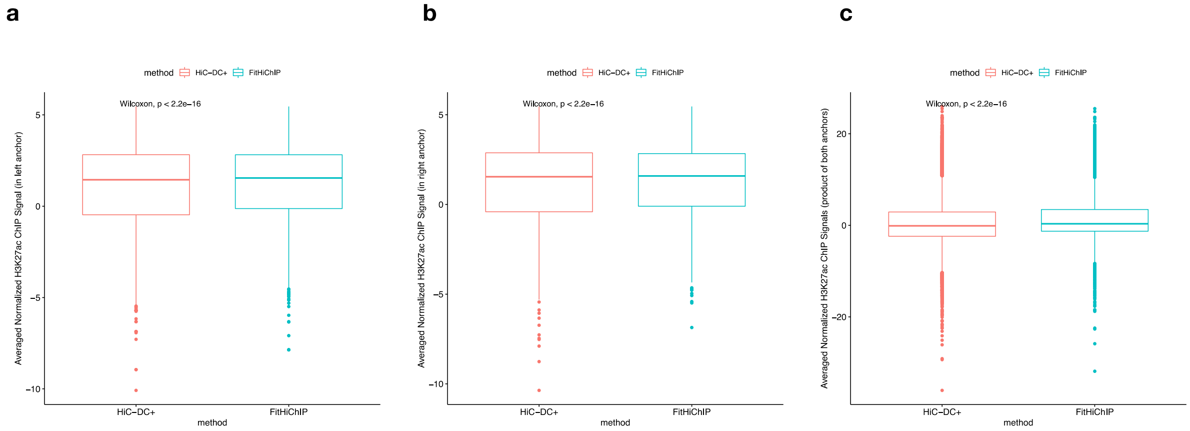

Supplementary Figure 10. Distribution of averaged normalized H3K27ac ChIP signal of the top 100,000 interactions identified by HiC-DC+ and FitHiChIP in K562 H3K27ac HiChIP (merged replicates of GSM2705043, GSM2705044, GSM2705045). ChIP signal (GSM733656) in the (**a**) left anchor, (**b**) the right anchor, and (**c**) the product of signal in both anchor (n=100,000 for both HiC-DC+ and FitHiChIP, and *P* values reported based on two-sided Wilcoxon tests). Centers of the boxes indicate median values, the lower and upper hinges correspond to the first and third quartiles, and the upper (lower) whiskers extend from the hinge to the largest (smallest) value no further than 1.5 times the distance between the first and third quartiles. Data beyond the end of the whiskers are plotted as individual dots.

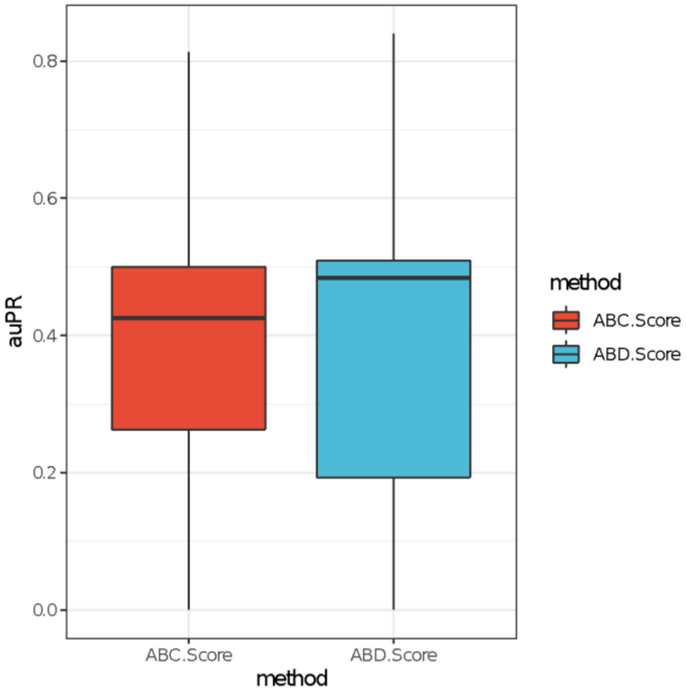

Supplementary Figure 11. Comparison of per gene auPR values between Activity-by-Contact and Activity-by-Distance methods using CRISPRi-FlowFISH data in K562 cells from Fulco et al.[4] (n=23 genes; Methods, Supplementary Note 2). Centers of the boxes indicate median values, the lower and upper hinges correspond to the first and third quartiles and the upper (lower) whiskers extend from the hinge to the largest (smallest) value no further than 1.5 times the distance between the first and third quartiles.

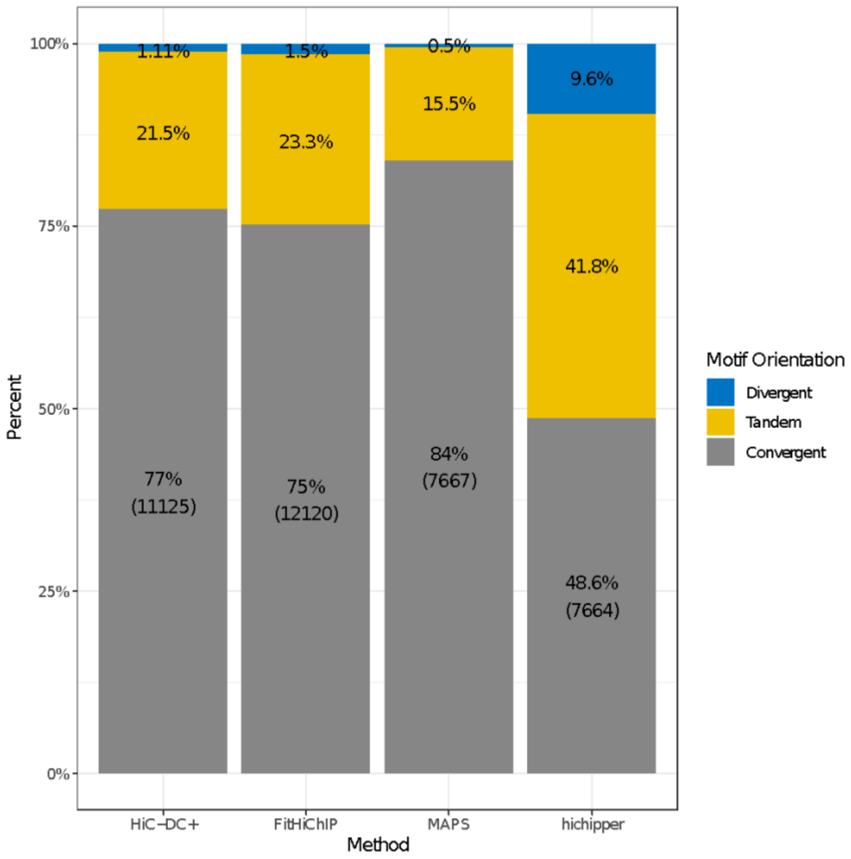

Supplementary Figure 12. CTCF motif orientations of GM12878 SMC1A HiChIP (merged replicates of GSM2138324, GSM2138325, GSM2138326, GSM2138327) loop anchors across different HiChIP interaction callers. We restricted the analysis to loops whose anchors both overlapped with CTCF motifs (Methods).

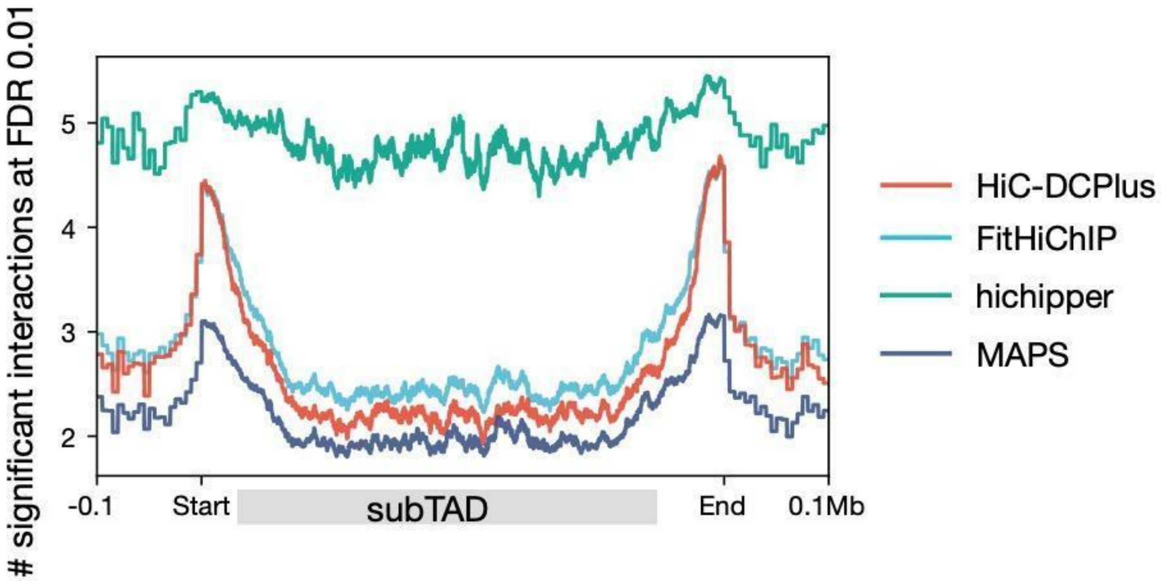

Supplementary Figure 13. Metaplot of the number of significant GM12878 SMC1A HiChIP (merged replicates of GSM2138324, GSM2138325, GSM2138326, GSM2138327) interactions (FDR < 0.01) anchored at each 5kb bin along GM12878 subTADs (GSE63525).

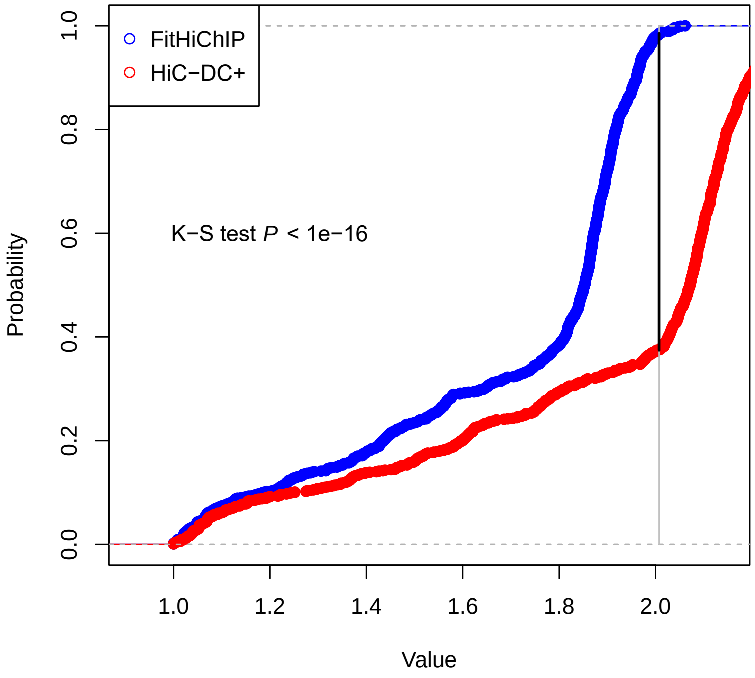

Supplementary Figure 14. CDF of enrichment of GM12878 SMC1A HiChIP (merged replicates of GSM2138324, GSM2138325, GSM2138326, GSM2138327) signal at subTAD boundaries with respect to interior of subTADs (GSE63525). Enrichment throughout the subTAD was estimated by dividing the average value at subTAD boundaries by average values within the subTAD used to generate the metaplot in Supplementary Fig. 13 and enrichment values for HiC-DC+ are found to be greater than that of FitHiChIP ($P < 1x10^{-16}$, one-sided K-S test).

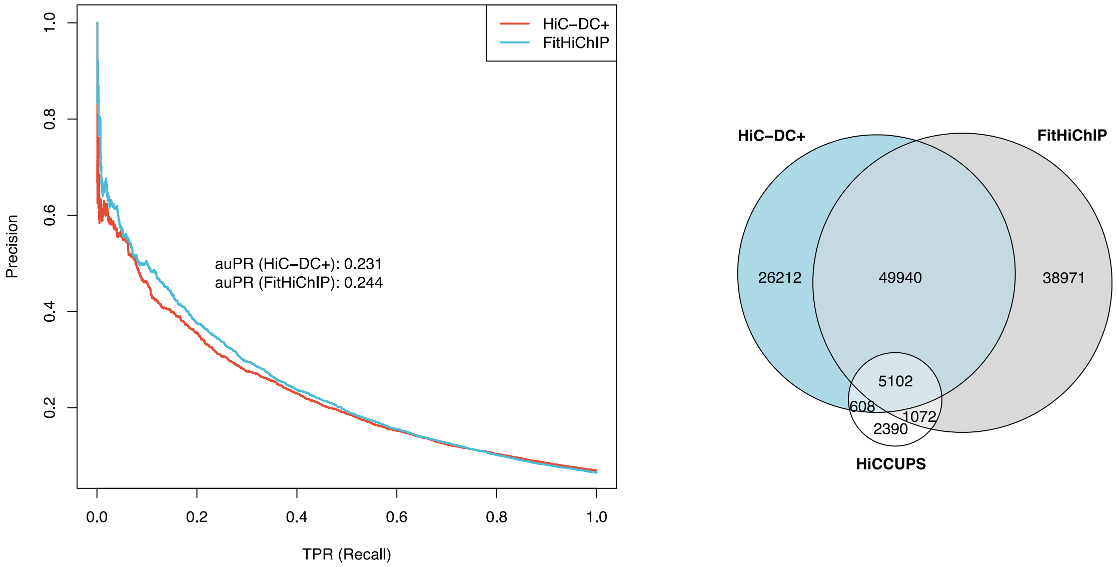

Supplementary Figure 15. Comparison of GM12878 SMC1A HiChIP (merged replicates of GSM2138324, GSM2138325, GSM2138326, GSM2138327) calls. **(Left)** Precision-recall curve for detection of HiCCUPS loops on GM12878 Hi-C (GSE63525, defined as ground truth) with HiC-DC+ applied to SMC1A HiChIP vs FitHiChIP applied to SMC1A HiChIP and SMC1A ChIP-seq in GM12878. **(Right)** Venn diagram of HiCCUPS calls, HiC-DC+ HiChIP calls at 1% FDR, and FitHiChIP calls at 1%FDR.

a b

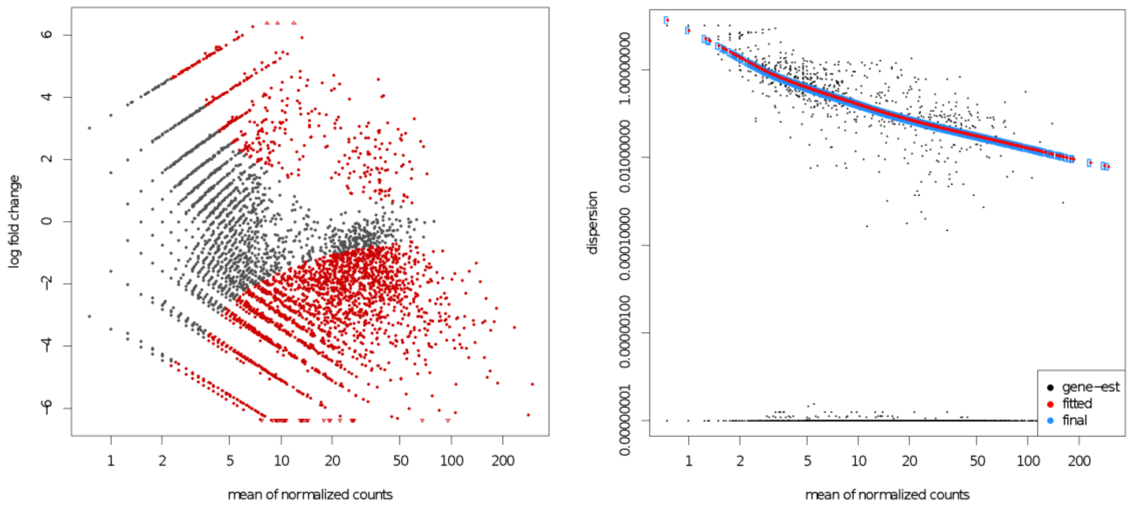

Supplementary Figure 16. Diagnostic plots for differential interaction calling. **a.** MA plot and **b.** plot of dispersion as a function of mean of normalized counts for differential interaction analysis of H3K27ac HiChIP for mESC (GSM3103921, GSM3103922) vs. MEF[5] (GSM3103923, GSM3103924) over chr2 at 5kb resolution.

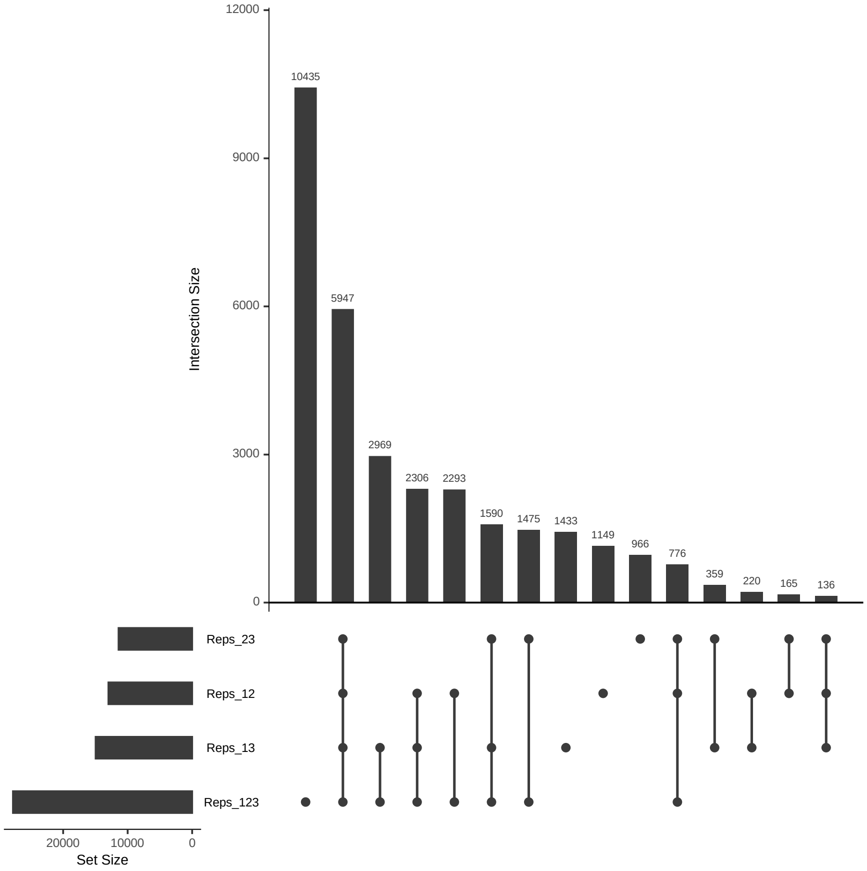

Supplementary Figure 17. Overlap among differential HiC-DC+ interactions called from different replicate combinations. We used Hi-C data with 4 replicates in mESC (4DNESDXUWBD9) and NPC (4DNESJ9SIAV5) from Bonev et al.[6] We found differential interactions between mESC and NPC using all three replicates (123), and all pairwise combinations of replicates (12, 13, and 23).

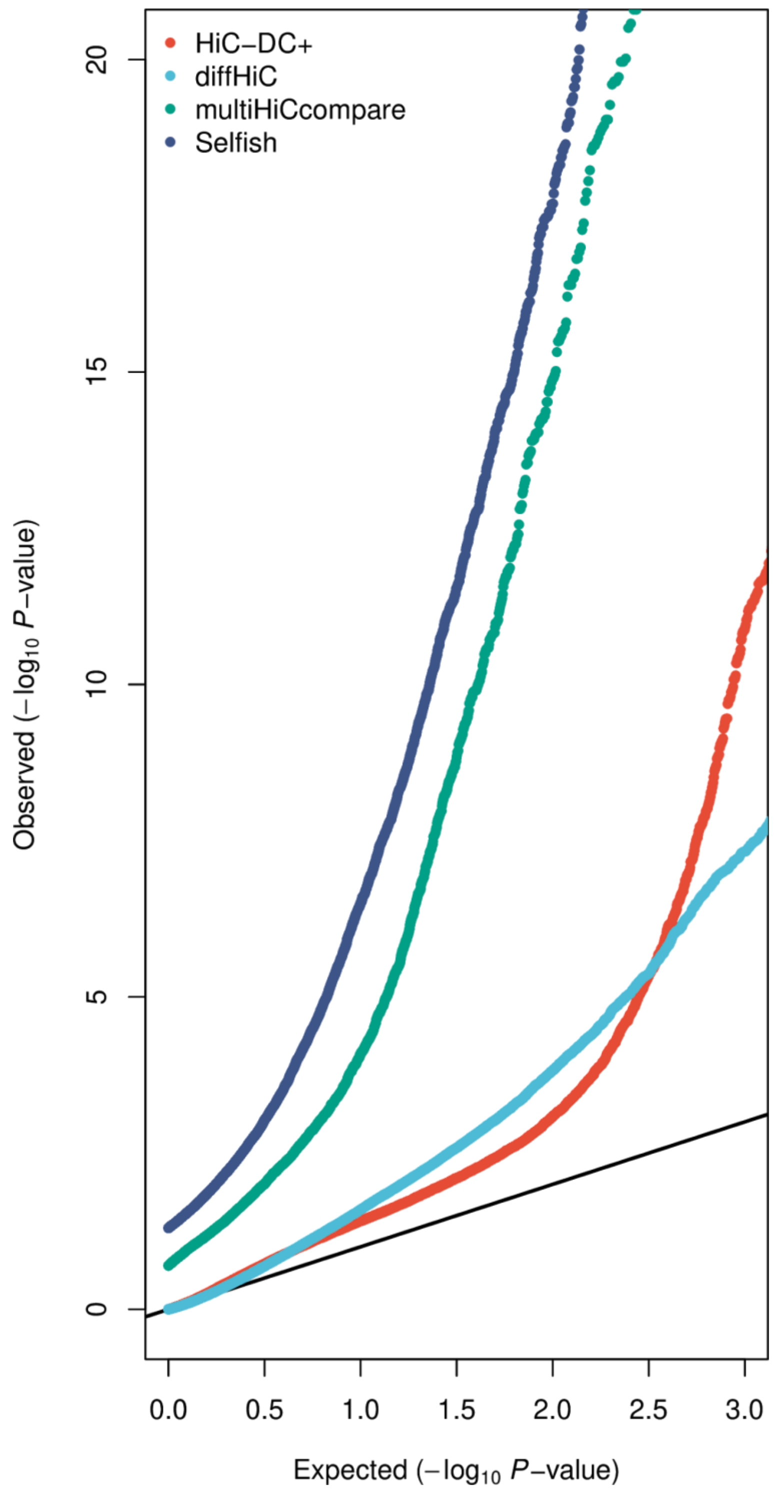

Supplementary Figure 18. Q-Q plots of differential Hi-C interaction calling methods on WAPL KO HAP1 (GSM2515800, GSM2515801, GSM2515802) vs. HAP1 (GSE74072) at 25kb resolution[7]. We restricted this analysis to interactions defined by HiC-DC+ (FDR < 0.1), as HiC-DC+ differential calling was run on this set of interactions instead of the whole genome.

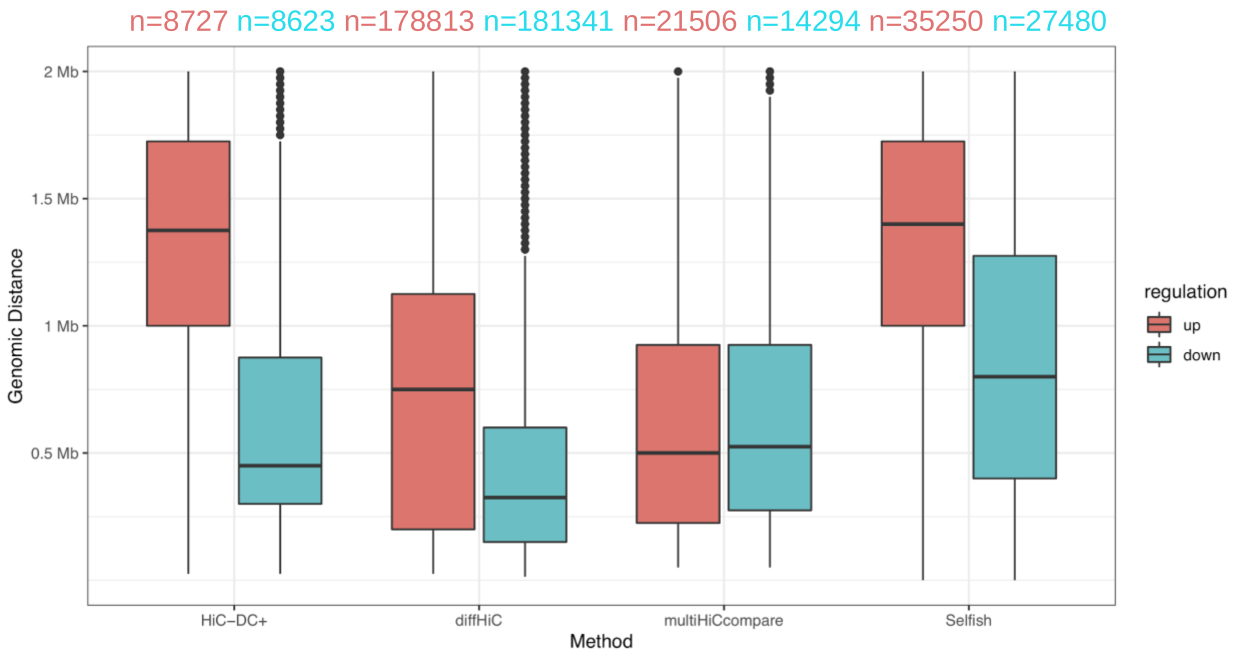

Supplementary Figure 19. Loop lengths of differential interactions (FDR < 0.05) called by HiC-DC+, diffHic, multiHiCcompare and Selfish on WAPL KO HAP1 (GSM2515800, GSM2515801, GSM2515802) vs. HAP1 Hi-C (GSE74072) at 25kb resolution[7]. We filtered out differential interactions with distances equal to 0 or more than 2Mb, and numbers of differential interactions found by each method at this distance range are provided at the top of the figure. Centers of the boxes indicate median values, the lower and upper hinges correspond to the first and third quartiles, and the upper (lower) whiskers extend from the hinge to the largest (smallest) value no further than 1.5 times the distance between the first and third quartiles. Data beyond the end of the whiskers are plotted as individual dots.

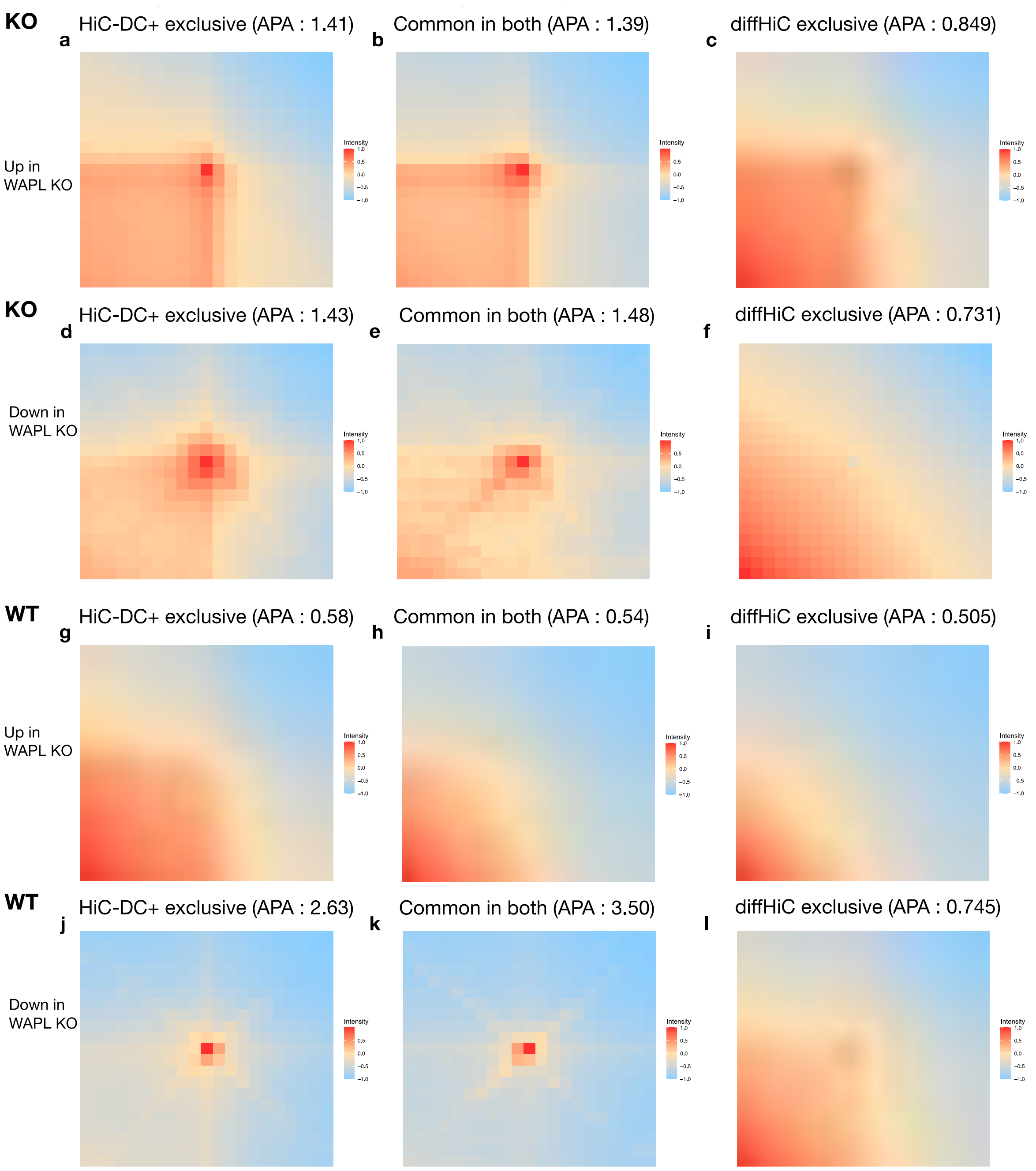

Supplementary Figure 20. APA plots for comparing differential Hi-C interactions called by HiC-DC+ and diffHic in WAPL KO (GSM2515800, GSM2515801, GSM2515802) and WT HAP1 (GSE74072) cells[7] (Methods). The first and the third rows show differentially gained interactions upon WAPL loss in WAPL KO and WT HAP1 cells, respectively; the second and the last rows show differentially lost interactions upon WAPL loss in WAPL KO and WT HAP1 cells, respectively. The first column shows differential interactions called exclusively by HiC-DC+; the second column shows shared differential interactions between HiC-DC+ and diffHic; the third column shows differential interactions exclusive to diffHic at 25kb. Number of interactions that passed APA filtering is: 4,124 for **(a,g)**; 3,354 for **(b,h)**; 87,114 for **(c,i)**; 1,863 for **(d,j)**; 895 for **(e,k)**; 32,647 for **(f,l).**

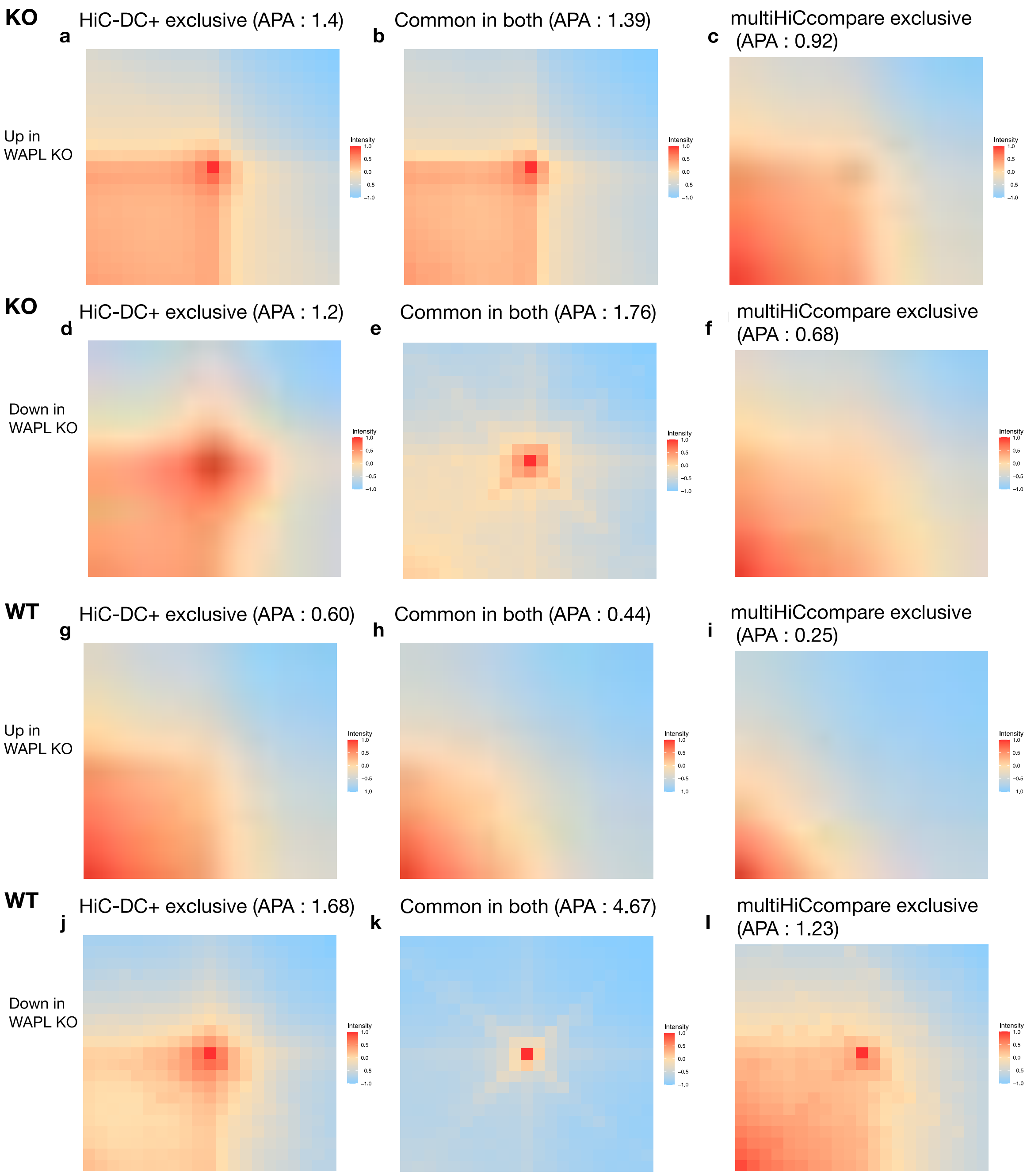

Supplementary Figure 21. APA plots for comparing differential Hi-C interactions called by HiC-DC+ and multiHiCcompare in WAPL KO (GSM2515800, GSM2515801, GSM2515802) and WT HAP1 (GSE74072) cells (Methods). The first and the third rows show differentially gained interactions upon WAPL loss in WAPL KO and WT HAP1 cells, respectively; the second and the last rows show differentially lost interactions upon WAPL loss in WAPL KO and WT HAP1 cells[7], respectively. The first column shows differential interactions called exclusively by HiC-DC+; the second column shows shared differential interactions between HiC-DC+ and multiHiCcompare; the third column shows differential interactions exclusive to multiHiCcompare at 25kb. Number of interactions that passed APA filtering is: 5,711 for **(a,g)**; 1,767 for **(b,h)**; 5,843 for **(c,i)**; 1,449 for **(d,j)**; 1,309 for **(e,k)**; 3,697 for **(f,l).**

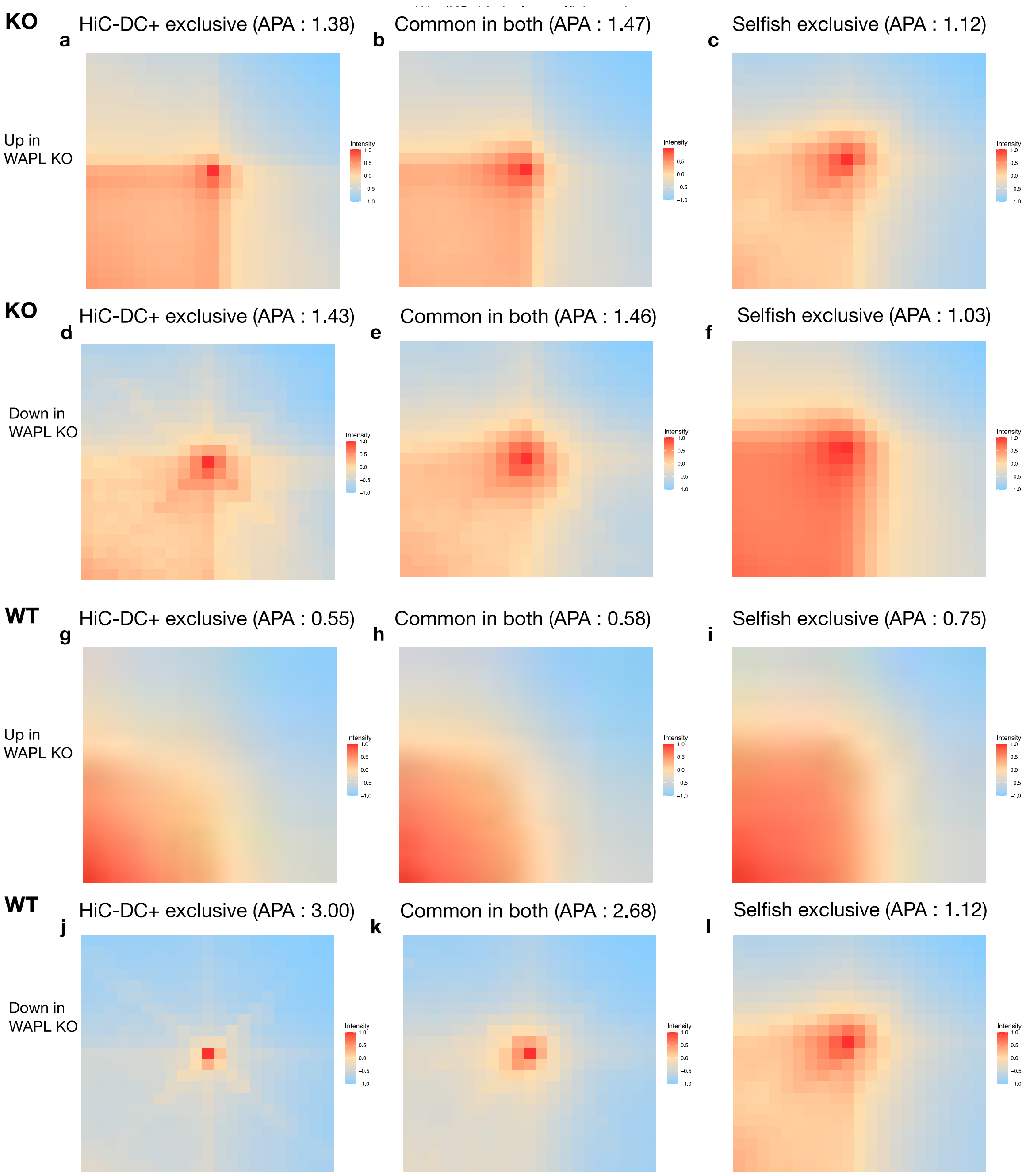

Supplementary Figure 22. APA plots for comparing differential Hi-C interactions called by HiC-DC+ and Selfish in WAPL KO (GSM2515800, GSM2515801, GSM2515802) and WT HAP1 (GSE74072) cells[7] (Methods). The first and the third rows show differentially gained interactions upon WAPL loss in WAPL KO and WT cells, respectively; the second and the last rows show differentially lost interactions upon WAPL loss in WAPL KO and WT HAP1 cells, respectively. The first column shows differential interactions called exclusively by HiC-DC+; the second column shows shared differential interactions between HiC-DC+ and Selfish; the third column shows differential interactions exclusive to Selfish at 25kb. Number of interactions that passed APA filtering is: 5,880 for **(a,g)**; 1,598 for **(b,h)**; 28,612 for **(c,i)**; 1,701 for **(d,j)**; 1,057 for **(e,k)**; 13,723 for **(f,l).**

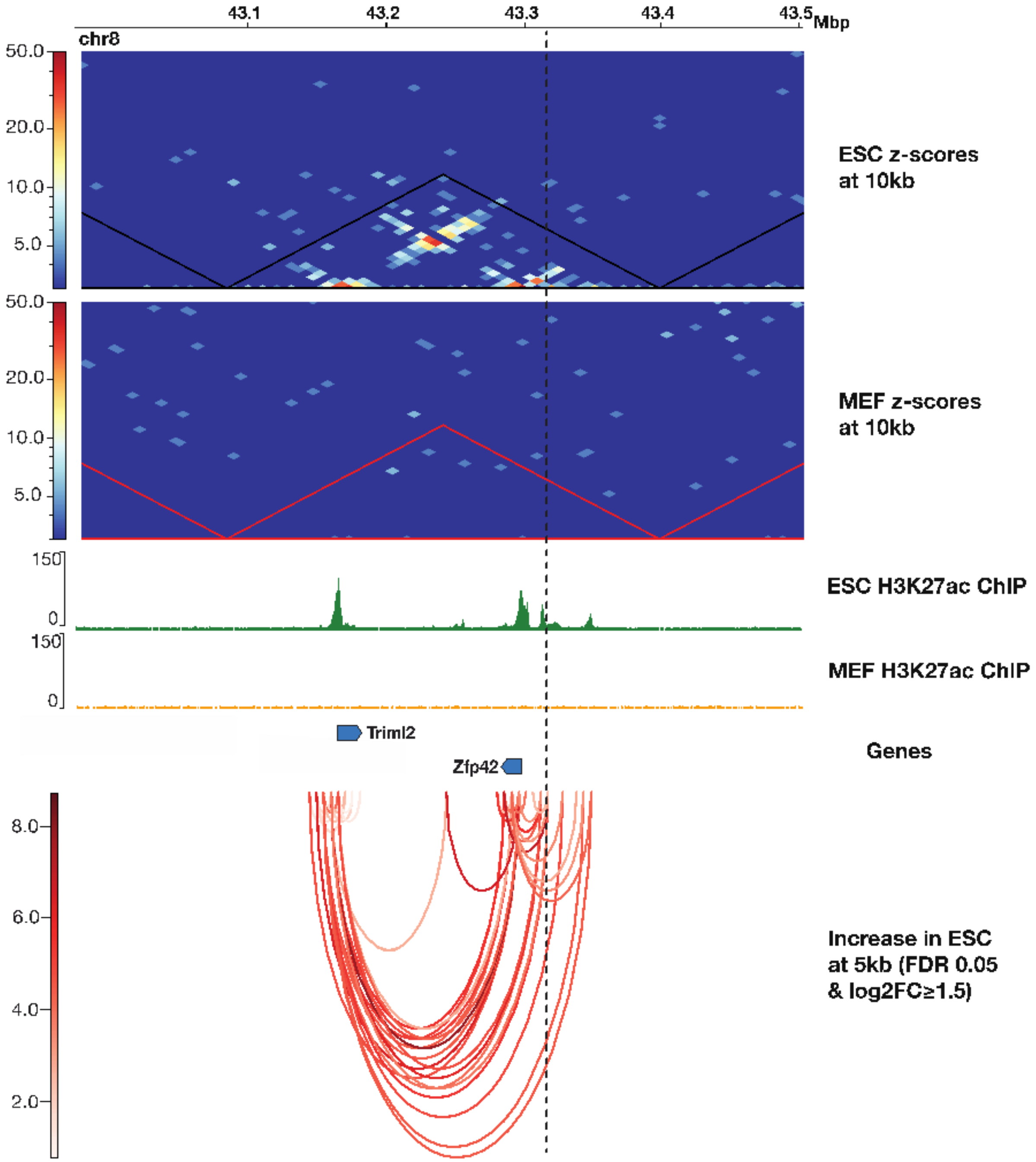

Supplementary Figure 23. HiC-DC+ detected mESC-specific H3K27ac HiChIP (replicates of GSM3103921, GSM3103922, GSM3103923, GSM3103924) interactions of an enhancer hub comprising *Zfp42* and *Triml2* at 5kb resolution.

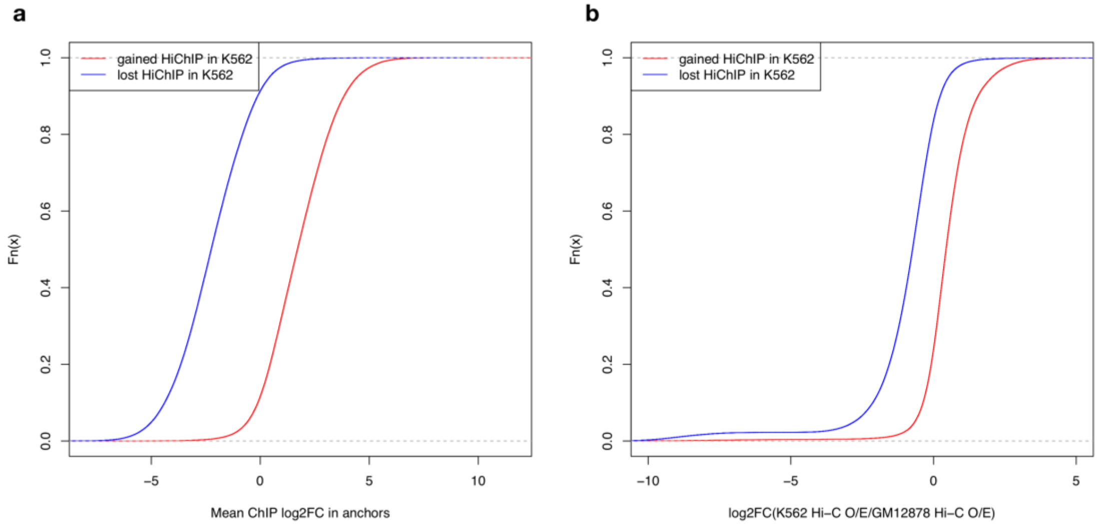

Supplementary Figure 24. CDF of change in ChIP-seq signal and Hi-C signal associated with differential HiChIP interactions (FDR < 0.05 and |logFC| >1) in K562 over GM12878. **a.** CDF of mean log fold change of ChIP signal (GSM733656 over GSM733771) across interaction bin anchors for differentially gained and lost HiChIP interactions in K562 (GSM2705043, GSM2705044, GSM2705045 over GSM2705041, GSM2705042) over GM12878 at 5kb; **b.** CDF of log fold change of HiC-DC+ normalized (O/E) Hi-C signal (GSM1551618, GSM1551619, GSM1551620, GSM1551621, GSM1551622, GSM1551623 over GSE63525) for differentially gained and lost HiChIP interactions in K562 over GM12878 at 5kb. Differential HiChIP interactions exhibited concordant change in ChIP-seq signal ($P < 1x10^{-16}$, one-sided K-S test) and change in Hi-C signal ($P < 1x10^{-16}$, one-sided K-S test).

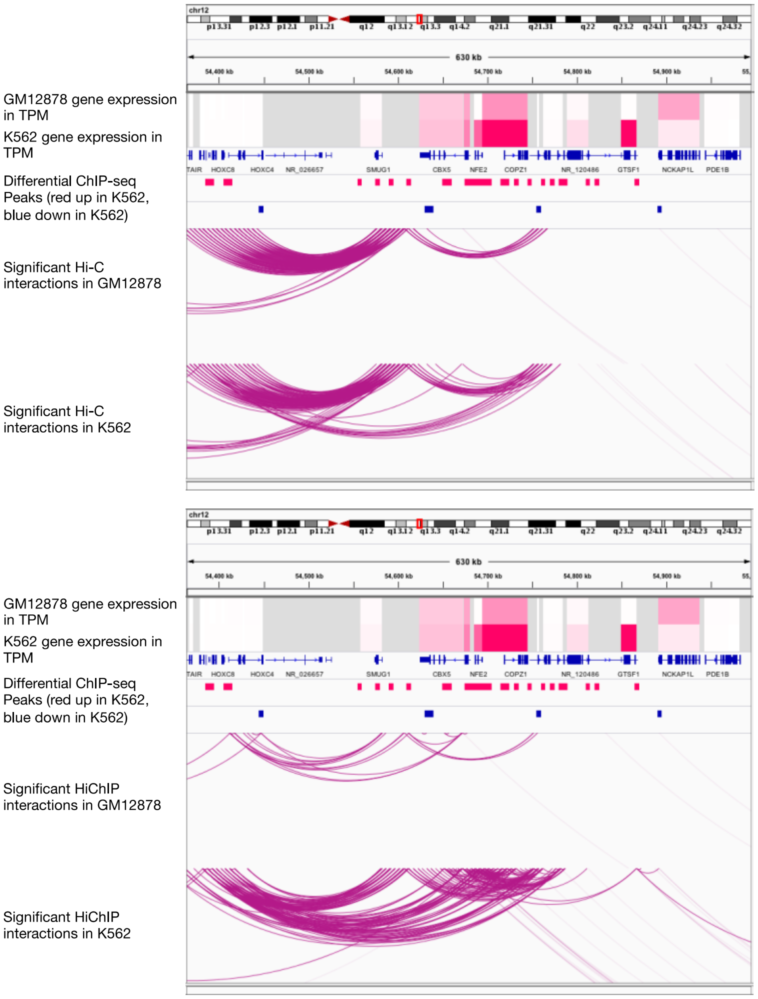

Supplementary Figure 25. IGV tracks of significant Hi-C (GSM1551618, GSM1551619, GSM1551620, GSM1551621, GSM1551622, GSM1551623 for K562, and GSE63525 for GM12878) and HiChIP (GSM2705043, GSM2705044, GSM2705045 for K562, and GSM2705041, GSM2705042 for GM12878) interactions around the up-regulated genes in K562 including *NFE2*, *COPZ1* and *GTSF1* showing HiChIP interactions (**bottom track**) change around this locus; while Hi-C interactions (**top track**) remain mostly stable. For gene expression, TPM values were illustrated as red being high, and gray being low. Gained differential ChIP-seq peaks in K562 (GSM733656) over GM12878 (GSM733771) were represented as red peaks, whereas lost ones were represented as blue.

a

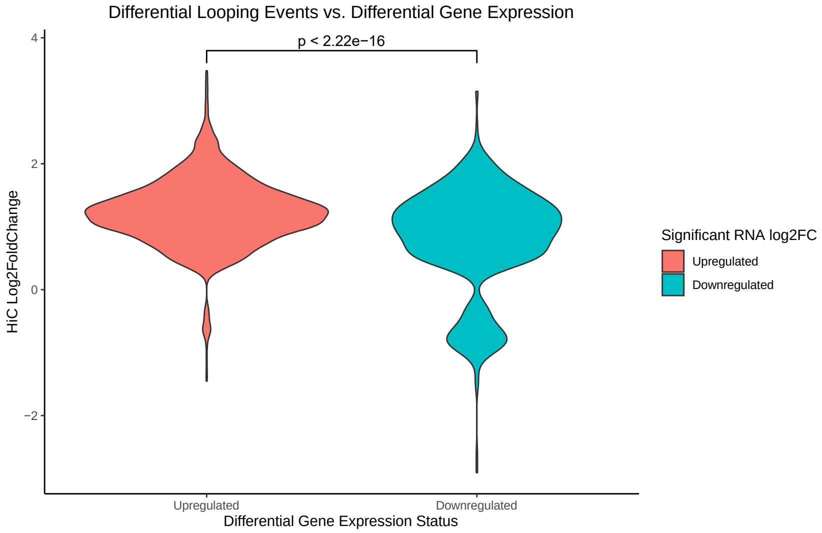

b

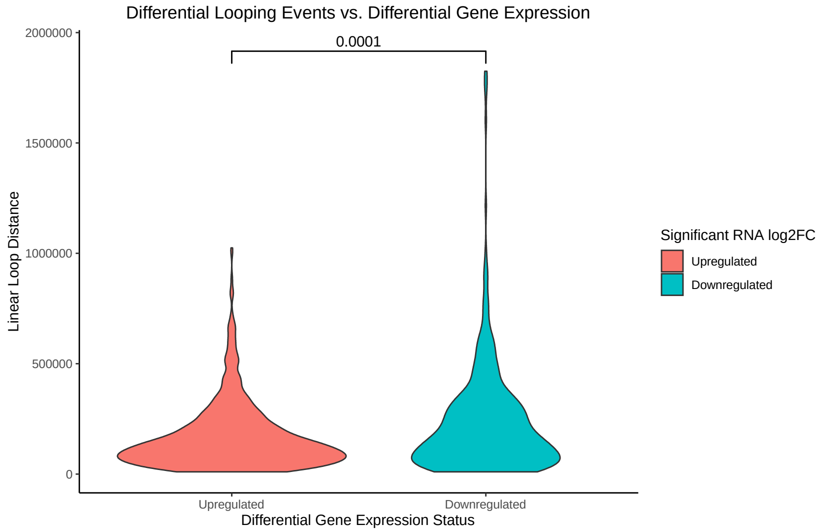

Supplementary Figure 26. Distribution of log fold changes (**a**) and distances (**b**) of significant differential Hi-C interactions (FDR < 0.05) with one anchor overlapping the promoter of a differentially expressed gene (FDR < 0.05) between THP-1 monocyte (PRJNA385337) and macrophages (PRJNA385337) at 5kb resolution. Upregulated genes are those whose expression increased in macrophages (GSM2599707, GSM2599708, GSM2599709, GSM2599710).

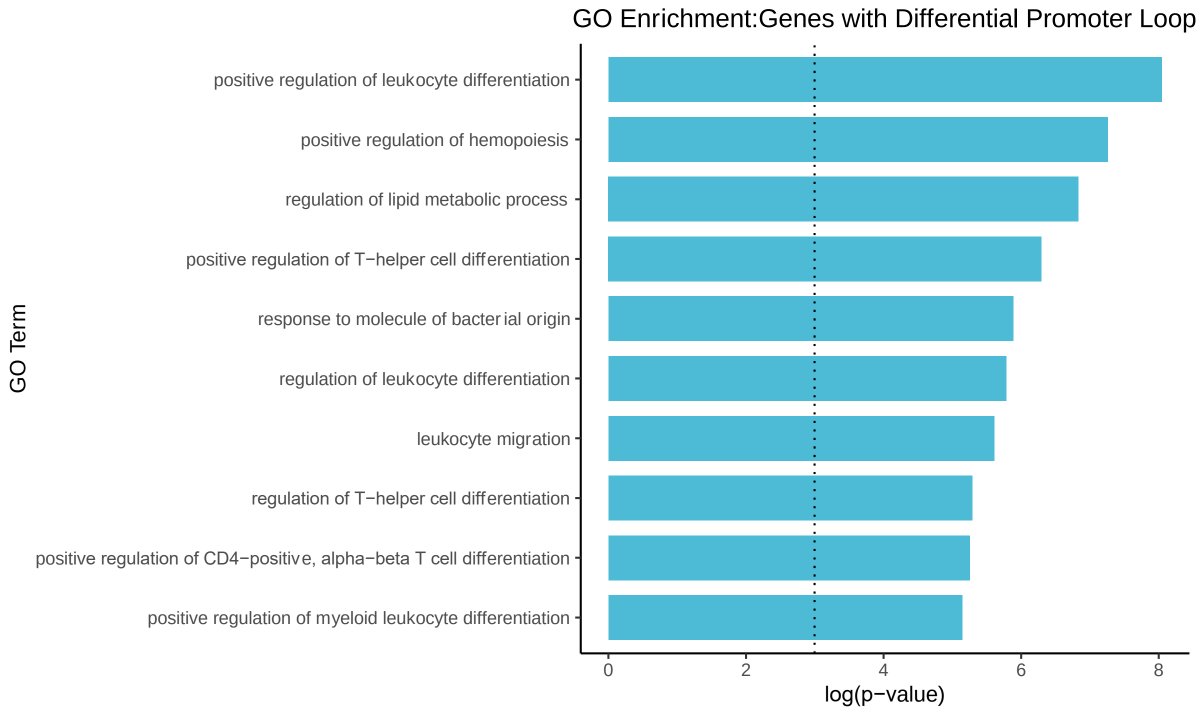

Supplementary Figure 27. GO enrichment analysis of genes with differential promoter-anchored loops (FDR < 0.05) in THP-1 macrophages (PRJNA385337) vs. THP-1 monocytes (PRJNA385337). We filtered results for significance (hypergeometric test, adjusted *P* < 0.05) while restricting to GO terms associated with at most 300 genes (Methods).

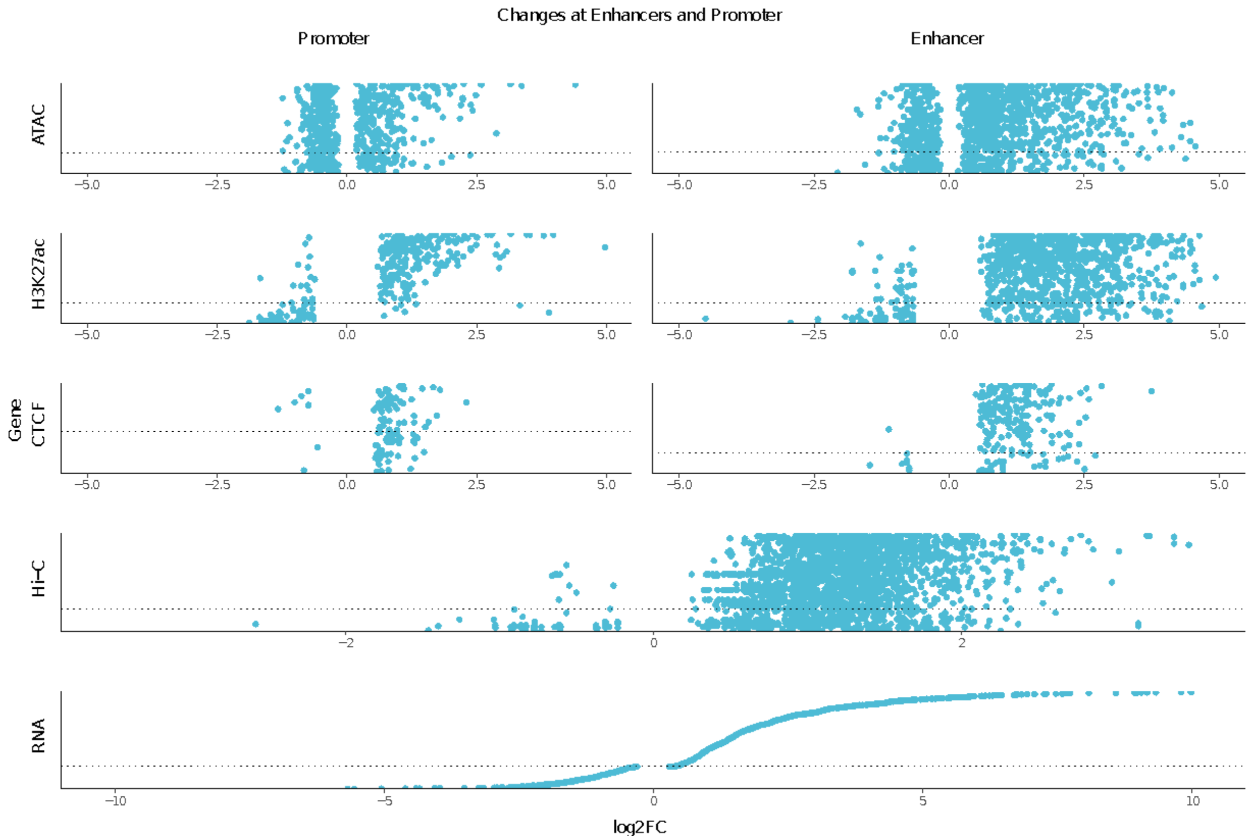

Supplementary Figure 28. Changes in ATAC-seq (GSM2544216, GSM2544217, GSM2544220, GSM2544221, GSM2544224, GSM2544225, GSM2544228, GSM2544229, GSM2544218, GSM2544219, GSM2544222, GSM2544223, GSM2544226, GSM2544227, GSM2544230, GSM2544231), H3K27ac (GSM2544236, GSM2544237, GSM2544238, GSM2544239), and CTCF ChIP-seq (GSM2544244, GSM2544245, GSM2544246, GSM2544247) at promoter and enhancer anchors of differential promoter-enhancer interactions (Methods) between THP-1 macrophages and monocytes at 5kb along with log fold changes of differential Hi-C (PRJNA385337) interactions and corresponding genes (GSM2599707, GSM2599708, GSM2599709, GSM2599710).

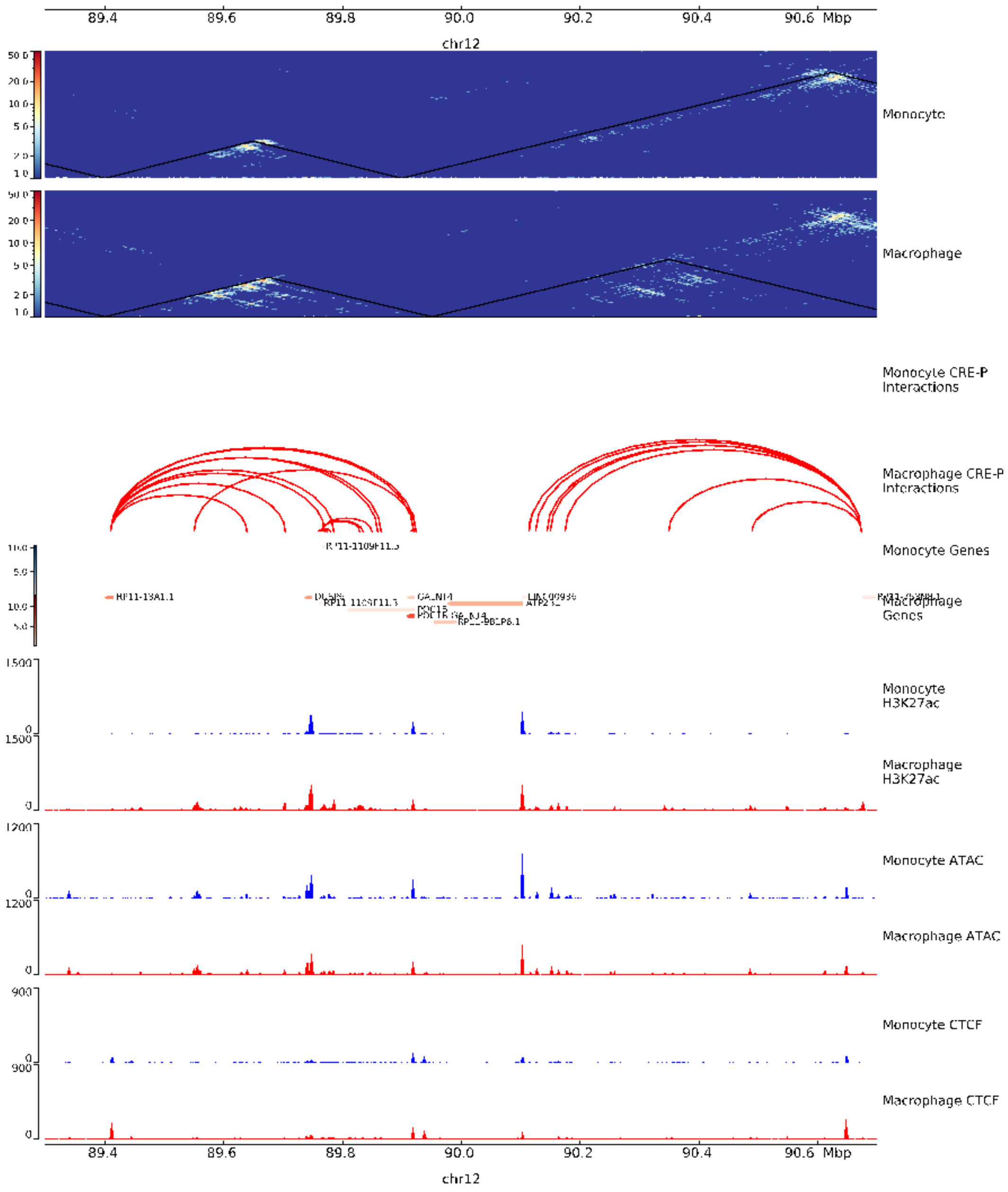

Supplemental Figure 29. Differential looping events at 5kb resolution around the *GALNT4* locus along with HiC-DC+ Z-score normalized Hi-C counts (PRJNA385337) (as heatmaps), log fold changes of differential genes (GSM2599707, GSM2599708, GSM2599709, GSM2599710), and CTCF (GSM2544244, GSM2544245, GSM2544246, GSM2544247), H3K27ac ChIP-seq (GSM2544236, GSM2544237, GSM2544238, GSM2544239) and ATAC-seq (GSM2544216, GSM2544217, GSM2544220, GSM2544221, GSM2544224, GSM2544225, GSM2544228, GSM2544229, GSM2544218, GSM2544219, GSM2544222, GSM2544223, GSM2544226, GSM2544227, GSM2544230, GSM2544231) signals in THP-1 macrophages and monocytes.

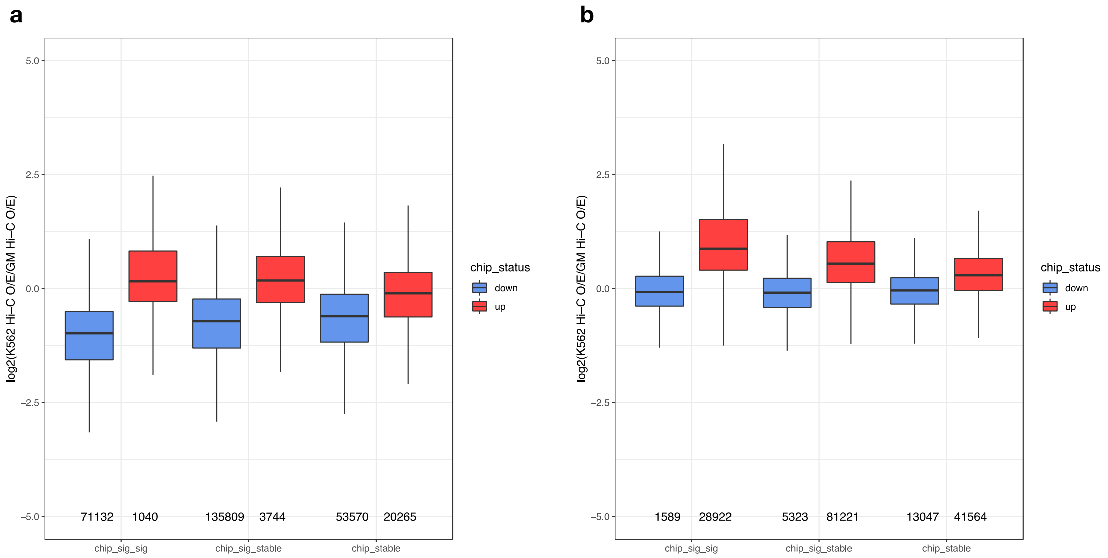

Supplementary Figure 30. Change in HiC-DC+ normalized Hi-C (O/E) counts in K562 (GSM1551618, GSM1551619, GSM1551620, GSM1551621, GSM1551622, GSM1551623) over GM12878 (GSE63525) in different categories (Supplementary Note 3) of **a.** lost and **b.** gained H3K27ac HiChIP interactions in K562 (GSM2705043, GSM2705044, GSM2705045) over GM12878 (GSM2705041, GSM2705042) at 5kb. ChIP-seq up or down status of an interaction is based on the average ChIP-seq log-fold change across peaks overlapping with the anchors of the interaction (GSM733656 for K562, and GSM733771 for GM12878). Numbers of interactions in each category are provided at the bottom part of the figure. Centers of the boxes indicate median values, the lower and upper hinges correspond to the first and third quartiles, and the upper (lower) whiskers extend from the hinge to the largest (smallest) value no further than 1.5 times the distance between the first and third quartiles.

Supplementary References

1. Mumbach, M.R. et al. HiChIP: efficient and sensitive analysis of protein-directed genome architecture. *Nat Methods* **13**, 919-922 (2016).
2. Rao, S.S. et al. A 3D map of the human genome at kilobase resolution reveals principles of chromatin looping. *Cell* **159**, 1665-1680 (2014).
3. Krietenstein, N. et al. Ultrastructural Details of Mammalian Chromosome Architecture. *Mol Cell* **78**, 554-565 e557 (2020).
4. Fulco, C.P. et al. Activity-by-contact model of enhancer-promoter regulation from thousands of CRISPR perturbations. *Nat Genet* **51**, 1664-1669 (2019).
5. Di Giammartino, D.C. et al. KLF4 is involved in the organization and regulation of pluripotency-associated three-dimensional enhancer networks. *Nat Cell Biol* **21**, 1179-1190 (2019).
6. Bonev, B. et al. Multiscale 3D Genome Rewiring during Mouse Neural Development. *Cell* **171**, 557-572 e524 (2017).
7. Haarhuis, J.H.I. et al. The Cohesin Release Factor WAPL Restricts Chromatin Loop Extension. *Cell* **169**, 693-707 e614 (2017).
