# Supplementary material for: HiC-DC+ enables systematic 3D interaction calls and differential analysis for Hi-C and HiChIP
Source: Nat Commun. 2021 Jun 7;12:3366. doi: 10.1038/s41467-021-23749-x (PMC8184932; doi:10.1038/s41467-021-23749-x)
Supplement: Supplementary file 3 — Description of Additional Supplementary Files [file 41467_2021_23749_MOESM3_ESM.pdf]

Description of Additional Supplementary Files

File Name: Supplementary Data 1
Description: Accession codes for the datasets used in the manuscript.
