# Supplementary material for: HiC-DC+ enables systematic 3D interaction calls and differential analysis for Hi-C and HiChIP
Source: Nat Commun. 2021 Jun 7;12:3366. doi: 10.1038/s41467-021-23749-x (PMC8184932; doi:10.1038/s41467-021-23749-x)
Supplement: Supplementary file 5 — Reporting Summary [file 41467_2021_23749_MOESM5_ESM.pdf]

nature research

Corresponding author(s): Christina S. Leslie

Last updated by author(s): May 5, 2021

Reporting Summary

Nature Research wishes to improve the reproducibility of the work that we publish. This form provides structure for consistency and transparency in reporting. For further information on Nature Research policies, see our Editorial Policies and the Editorial Policy Checklist.

Statistics

For all statistical analyses, confirm that the following items are present in the figure legend, table legend, main text, or Methods section.

n/a	Confirmed	
☐	☒	The exact sample size (*n*) for each experimental group/condition, given as a discrete number and unit of measurement
☐	☒	A statement on whether measurements were taken from distinct samples or whether the same sample was measured repeatedly
☐	☒	The statistical test(s) used AND whether they are one- or two-sided<br>*Only common tests should be described solely by name; describe more complex techniques in the Methods section.*
☒	☐	A description of all covariates tested
☐	☒	A description of any assumptions or corrections, such as tests of normality and adjustment for multiple comparisons
☐	☒	A full description of the statistical parameters including central tendency (e.g. means) or other basic estimates (e.g. regression coefficient) AND variation (e.g. standard deviation) or associated estimates of uncertainty (e.g. confidence intervals)
☐	☒	For null hypothesis testing, the test statistic (e.g. *F*, *t*, *r*) with confidence intervals, effect sizes, degrees of freedom and *P* value noted<br>*Give P values as exact values whenever suitable.*
☒	☐	For Bayesian analysis, information on the choice of priors and Markov chain Monte Carlo settings
☒	☐	For hierarchical and complex designs, identification of the appropriate level for tests and full reporting of outcomes
☒	☐	Estimates of effect sizes (e.g. Cohen's *d*, Pearson's *r*), indicating how they were calculated

Our web collection on statistics for biologists contains articles on many of the points above.

Software and code

Policy information about availability of computer code

Data collection	We collected publicly available data from GEO, ENCODE and 4DN repositories. We did not use any tools to download data from ENCODE or 4DN. To download fastq files from GEO, we used fastq-dump (v_2.9.1-1).
Data analysis	The tool that we developed is available at https://bitbucket.org/leslielab/hicdcplus.<br><br>The tools that we used to analyze Hi-C and HiChIP data are HiC-Pro pipeline (v_2.11.1), Juicer pipeline (v_1.7.6), TopDom (v_0.0.2), MAPS (v_1.1.0), FitHiChIP (v_8.1.0), hichipper (v_0.7.0), diffloop (v_1.16.019), multiHiCcompare (v_1.6.0), Selfish (v_1.10.2), diffHic (v_1.14.0), GREAT (v_4.0.4).<br><br>The tools that we used to analyze ATAC-seq and ChIP-seq data are BWA (v_0.7.17-r1188), SAMtools (v_1.9), Picard tools (v_2.18.16), MACS2 (v_2.1.2), IDR (v_2.0.3), featureCounts (v_1.6.4), DESeq2 (v_1.24.0), Bedtools genomeCoverageBed (v_2.27.1), UCSC bedgraph2bigwig (v_4), cutadapt (v_2.3), Bowtie2 (v_2.3.4.3).<br><br>For visualization of metaplots, and Hi-C tracks, we used deepTools (v_3.1.1) and Juicebox (v_1.9.8).

For manuscripts utilizing custom algorithms or software that are central to the research but not yet described in published literature, software must be made available to editors and reviewers. We strongly encourage code deposition in a community repository (e.g. GitHub). See the Nature Research guidelines for submitting code & software for further information.

Data

Policy information about availability of data

All manuscripts must include a data availability statement. This statement should provide the following information, where applicable:

- Accession codes, unique identifiers, or web links for publicly available datasets
- A list of figures that have associated raw data
- A description of any restrictions on data availability

We used publicly available data reposited to ENCODE portal, 4DN and GEO, and provided the accession codes in the Supplementary Table 7.

Field-specific reporting

Please select the one below that is the best fit for your research. If you are not sure, read the appropriate sections before making your selection.

[x] Life sciences [ ] Behavioural & social sciences [ ] Ecological, evolutionary & environmental sciences

For a reference copy of the document with all sections, see nature.com/documents/nr-reporting-summary-flat.pdf

Life sciences study design

All studies must disclose on these points even when the disclosure is negative.

Sample size	We used publicly available datasets without excluding any samples.
Data exclusions	We did not exclude any data that is publicly available for the cell or assay of interest.
Replication	For the publicly available datasets with replicates, we assessed our model's reproducibility by measuring the correlation and also finding the overlap between the interactions detected in each replicate.
Randomization	We did not design the experiments, so this is not relevant.
Blinding	We used publicly available data, so this is not relevant.

Reporting for specific materials, systems and methods

We require information from authors about some types of materials, experimental systems and methods used in many studies. Here, indicate whether each material, system or method listed is relevant to your study. If you are not sure if a list item applies to your research, read the appropriate section before selecting a response.

Materials & experimental systems

n/a	Involved in the study	
[x]	[ ]	Antibodies
[x]	[ ]	Eukaryotic cell lines
[x]	[ ]	Palaeontology and archaeology
[x]	[ ]	Animals and other organisms
[x]	[ ]	Human research participants
[x]	[ ]	Clinical data
[x]	[ ]	Dual use research of concern

Methods

n/a	Involved in the study	
[x]	[ ]	ChIP-seq
[x]	[ ]	Flow cytometry
[x]	[ ]	MRI-based neuroimaging
